# Metabolic and molecular analysis of nonuniform anthocyanin pigmentation in tomato fruit under high light

**DOI:** 10.1038/s41438-019-0138-2

**Published:** 2019-05-01

**Authors:** Yanjie Zhang, Yan Li, Wanping Li, Zongli Hu, Xiaohui Yu, Yun Tu, Min Zhang, Jinyong Huang, Guoping Chen

**Affiliations:** 1 Bioengineering College, Chongqing University, 400030 Chongqing, People’s Republic of China; 2 School of Agricultural Sciences, Zhengzhou University, 450001 Zhengzhou, People’s Republic of China

**Keywords:** Secondary metabolism, Plant physiology

## Abstract

Pigment intensity and patterns are important factors that determine the nutritional and market values of tomato fruits. The acropetal manner of light-dependent anthocyanin accumulation with the highest levels at the stem end of the fruit makes *Pro35S:BrTT8* tomato plants an ideal system for investigating the effects of light intensity on anthocyanin biosynthesis. Extensive transcript analyses indicate that anthocyanin pigmentation in *Pro35S:BrTT8* plants under high light might be coordinately regulated by the exogenous protein BrTT8 and endogenous proteins SlAN2 and SlMYBL2. Furthermore, yeast two-hybrid assays showed that BrTT8 could interact efficiently with SlAN2, SlMYBL2, and SlAN11. Moreover, the physical interaction between BrTT8 and SlAN2 was validated by FRET. Simultaneous overexpression of SlAN2 and BrTT8 activated significant anthocyanin biosynthesis in infiltrated tobacco leaves. In addition, the ability of SlMYBL2 to suppress anthocyanin accumulation was also demonstrated in infiltrated tobacco leaves. Altogether, these results prove that tissue-specific assemblage of the heterogeneous MYB-bHLH-WD40 complex consisting of SlAN2, BrTT8 and SlAN11 triggers nonuniform anthocyanin accumulation in tomato fruit under high light. Additionally, it is proposed that a negative-feedback loop fulfilled by SlMYBL2 also participates in the regulation of anthocyanin production.

## Metabolism: the protein regulators of nutrient accumulation

Protein regulators of anthocyanin production have been identified in tomato and tobacco plants. Endogenously produced anthocyanins confer protective benefits to plants, and there is growing evidence of their health benefits to humans. Researchers from China, led by Chongqing University’s Guoping Chen, analyzed tobacco and genetically modified tomato plants grown under high- and low-light conditions to discern the cellular processes modulating anthocyanin production. The team found a number of interacting metabolites that promoted anthocyanin, in particular a complex of three proteins, and evidence of a negative-feedback loop to prevent over-accumulation. These results provide insight into the biosynthesis of plant nutrients, could inform future efforts to genetically engineer more nutritious and valuable fruit, and also validates the team’s mutant tomato plant as a strong model to study anthocyanin production.

## Highlight

- High-light-induced nonuniform anthocyanin pigmentation in fruits of *Pro35S:BrTT8* tomato plants is due to region-specific assemblage of a functional MBW protein complex consisting of BrTT8, SlAN2 and SlAN11.

## Introduction

Anthocyanins are the main water-soluble pigments widespread in many flowering plants. The red, blue, and purple colors found in various plant tissues, including flowers, leaves, fruits, and seeds, are mostly attributed to the accumulation of anthocyanins^1–3^. In addition to their well-known physiological functions of serving as pollinator and seed disperser attractants, anthocyanins also play essential roles in protecting plant tissues from being damaged by high light, UV radiation, cold, drought, nutrient deficiency, and pathogen attack^4^; anthocyanins also act as important health-promoting supplements in the human daily diet. A growing amount of evidence has shown that regular intake of anthocyanins is associated with a lower risk of artherosclerosis and related diseases due to their ability to inhibit the oxidation of low-density lipids^5^. The health-promoting effects of anthocyanins are considered to be closely linked to high antioxidant activities and the capacity to eliminate reactive oxygen species^6–8^. However, recent studies have shown that anthocyanins and related derivatives are involved in modulating signaling pathways in mammalian cells^9^. In addition, the effects of anthocyanins on the prevention of cancer and some other chronic illnesses have been demonstrated^10,11^.

**Fig. 1:**
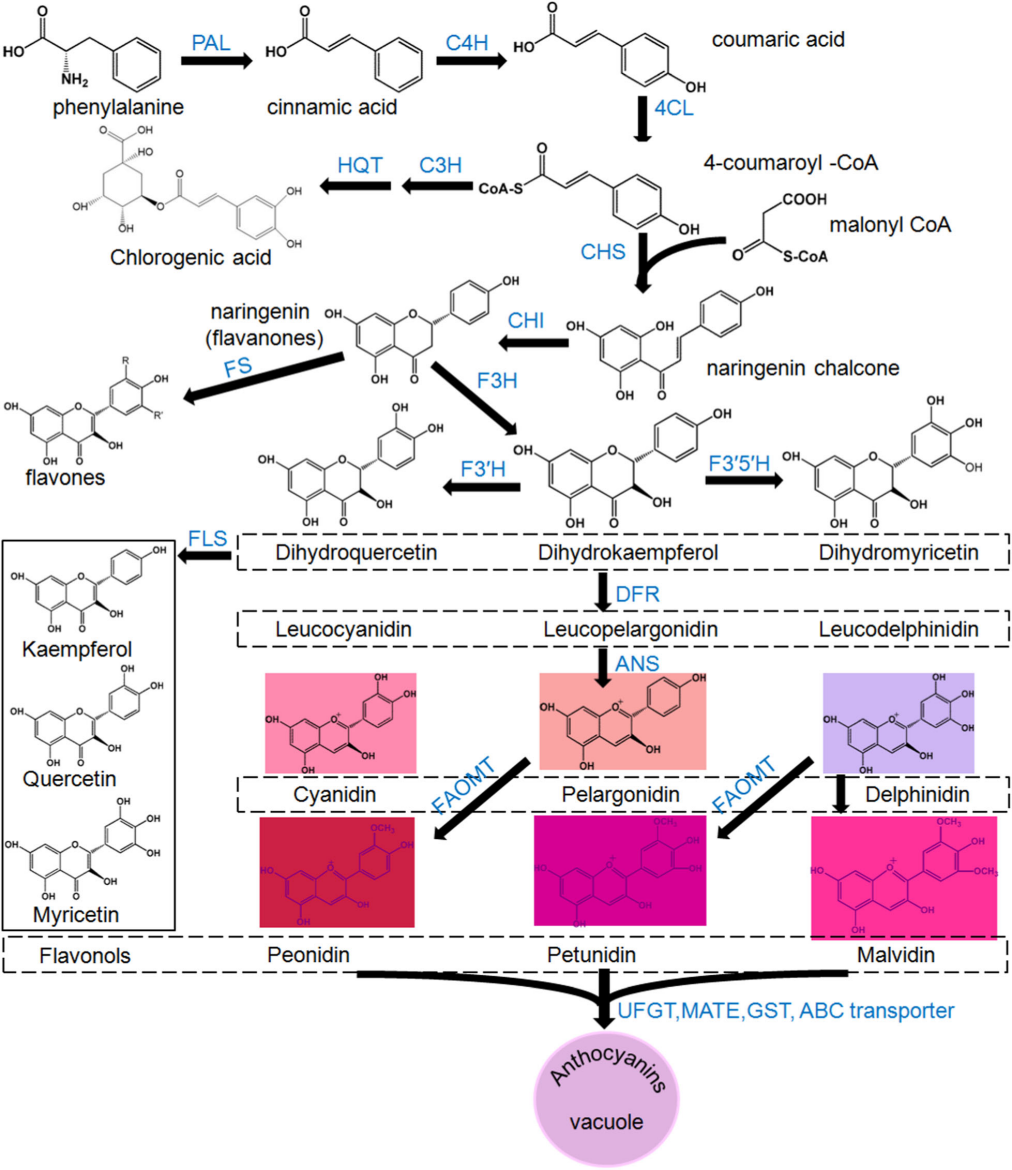
Schematic representation of the phenylpropane pathway

As the main subclass of phenylpropanoid metabolites, anthocyanins employ a biosynthetic pathway that has been well characterized (Fig. 1). The synthesis of cinnamic acid catalyzed by phenylalanine ammonia-lyase (PAL) represents the initial reaction step of the phenylpropanoid pathway^12^. Chalcone synthase (CHS) catalyzes the condensation reaction with 4-coumaroyl CoA and malonyl CoA as substrates to produce naringenin chalcone, the entry molecule of the flavonoid pathway. 4-Coumaroyl CoA can be esterified with quinic acid by hydroxycinnamoyl CoA-quinate transferase (HQT), leading to the production of chlorogenic acid (CGA), a typical phenolic acid widespread in Solanaceae plants. The basic skeleton of all flavonoids (including anthocyanins, chalcones, flavones, flavonols, flavandiols, and condensed tannins) consists of three aromatic rings generated by CHS and CHI. F3H catalyzes the oxidation of the central ring of naringenin, yielding dihydrokaempferol. Moreover, dihydrokaempferol can be further hydroxylated on the 3′ or 5′ position of the B-ring by flavonoid 3′-hydroxylase (F3′H) and/or flavonoid 3′ 5′-hydroxylase (F3′5′H), resulting in the production of dihydroquercetin and dihydromyricetin, respectively^2^. Additionally, cyanidin and delphinidin can be further methylated at the 3′ or 5′ position of the B-ring by flavonoid 3′, 5′-methyltransferase (FAOMT), resulting in the production of peonidin, petunidin and malvidin, respectively. Unstable anthocyanidins are modified mostly by glycosylation and converted into stable forms, namely, anthocyanins^2^. Then, the final productions are transported into vacuoles coordinately by multidrug and toxic compound extrusion (MATE) transporters, glutathione S-transferase (GST) or ABC transporters^13,14^. Except for anthocyanins, chalcones and CGA, flavonols and flavones can also be produced in the branch pathway of phenylpropanoid metabolism.

The biosynthesis of phenylalanine-derived compounds, especially flavonoids, is mainly regulated by MYB proteins, which constitute the largest class of modulators for secondary metabolism^15,16^. A total of 339 and 230 MYB proteins were characterized in *Arabidopsis thaliana* and rice, respectively^17^. Since structurally similar R2R3-MYB TFs were able to trigger identical target genes by binding with the same *cis* elements in promoters, functional redundancy for these MYB proteins has often been indicated^18,19^. By analyzing mutant plants with abnormal levels of anthocyanin production, many modulators were cloned and characterized, including basic helix–loop–helix (bHLH) TFs, WD40 repeat (WDR) proteins, basic Leucine Zipper (bZIP) TFs, WRKY TFs, MADS-box TFs and SQUAMOSA PROMOTER BINDING PROTEIN (SPL) TFs^20–23^. Further studies prove that structural genes catalyzing the late step reactions of the anthocyanin pathway are regulated directly by a ternary protein complex consisting of R2R3-MYB TFs (like PAP1, PAP2 MYB113, and MYB114 in *Arabidopsis*), bHLH TFs (like TT8, GL3, and EGL3), and WD40 repeat proteins (such as TTG1)^21,24,25^.

Among all the regulators mentioned above, MYB and bHLH proteins are thought to be the key factors that regulate spatial and temporal anthocyanin accumulation in plants^9,17,26^. It has been demonstrated that AtMYB4, an R2R3-MYB repressor, could repress the transcription of target gene *C4H* by a special motif in the C-terminal domain^27^. In subsequent studies, several anthocyanin biosynthesis repressors from the R2R3 and R3-MYB subfamilies have been identified and characterized. FaMYB1 and PhMYB27, belonging to the R2R3-MYB subfamily, have been identified as transcriptional repressors in strawberry^28^. In addition, small R3-MYB transcription factors that inhibit anthocyanin biosynthesis by acting as competitive inhibitors of the R2R3-MYB activators have also been cloned^29–31^. In *Arabidopsis*, CAPRICE (CPC), an R3-MYB protein was identified as a determinant of cell differentiation at first and was proved to be a transcriptional repressor in anthocyanin biosynthesis in transgenic tobacco and tomato plants in further studies^29,31^. In addition, *SlTRY*, an ortholog gene of *TRYPTICHON* (*TRY*), was identified, and the ectopic expression of *SlTRY* significantly reduced anthocyanin accumulation in transgenic *Arabidopsis*^31^. In the latest study, a 4-bp insertion in the coding region of the *atv* (*atroviolacium*) locus encoding a putative R3-MYB repressor significantly enhanced anthocyanin accumulation in tomato fruits^32,33^.

Except for endogenous signals, exogenous stimulation factors, such as high light, drought, cold and phosphorus deficiency, operate coordinately to accurately regulate the anthocyanin pathway^34–37^. In previous studies, several bHLH proteins have been introduced into *Arabidopsis*, tomato, *Nicotiana* and petunia for functional analysis in anthocyanin biosynthesis regulation^30,38–41^. In addition, light-induced anthocyanin accumulation was also carefully examined in the vegetative tissues of *Pro35S:ZmLc* petunia plants^35^. Moreover, *SlTT8* (also named *SlAN1* and *SlAH*) was shown to participate in the regulation of anthocyanin biosynthesis and is induced by low temperature^32,41,42^. However, high-light-induced anthocyanin pigmentation in tomato fruits has not been reported or studied. *Pro35S:BrTT8* plants created in our previous work displayed abundant anthocyanin accumulation in both vegetative and fruit tissues when grown in natural high-light conditions (open field) but remained anthocyaninless under low-light conditions (greenhouse)^44^. Interestingly, anthocyanin accumulates in a unique pattern in *Pro35S:BrTT8* fruits under natural high-light conditions. As tomato (*Solanum lycopersicum* L.) is an ideal model system for studying fleshy fruit development^43^. *Pro35S:BrTT8* tomato plants serve as an excellent model system for the study of nonuniform anthocyanin pigmentation in fruits exposed to high light.

## Materials and methods

### Chemicals and reagents

Ultrapure water was prepared by a Milli-Q purification system. HPLC grade methanol, acetonitrile, acetic acid, formic acid, neochlorogenic acid, chlorogenic acid, cryptochlorogenic acid, kaempferol, and delphinidin-3-O-rutinoside were purchased from Sigma-Aldrich.

### Plant material and growth conditions

Diploid tomato plants (*Solanum lycopersicum* Mill. cv Ailsa Craig, AC) were generously provided by Dr. Donald Grierson of the School of Biosciences, University of Nottingham, UK^44^. The *Pro35S:BrTT8* transgenic tomato seeds were generated from the primary transformants of AC in our previous article^45^. In this study, the existing photoperiod was 16 h, and the temperature in the greenhouse was maintained between 20 and 28 °C. In addition, the relative humidity was approximately 60%. Plants were cultured under low-light conditions to inhibit anthocyanin accumulation until they exhibited approximately six nodes. All of the axillary buds were cut to prevent a branched architecture, which may result in self-shading effects. Six replicates of both AC and *Pro35S:BrTT8* plants were transferred to either artificial low-light or high-light conditions. The seedlings were maintained for 10 days under each light treatment. After that, leaves were sampled and frozen immediately in liquid nitrogen and kept at −80 °C for pigment extraction and RNA isolation.

In the following experiments investigating nonuniform anthocyanin pigmentation in *Pro35S:BrTT8* fruits, 18 homozygous (six individuals for each independent transgenic line) plants and wild-type plants were grown in a greenhouse under artificial high-light conditions. Fruits were covered by translucent plastic bags after pollination to simulate low-light conditions. Epicarps and mesocarps were removed and sampled by hand, frozen immediately in liquid nitrogen and kept at −80 °C for pigment extraction and RNA isolation.

### Light treatments

The high-light treatment was provided by supplementing normal light with a metal-halide lamp (Philips) under a short-day (8 h of illumination) photoperiod, and plants were arranged randomly to prevent shading. The light level varied between 50 and 100 μmol m^−2^ s^−1^ in the low-light conditions, while the light intensity was constant at 700 μmol m^−2^s^−1^ in the artificial high-light conditions. Unless otherwise stated, samples were all collected at the end of the light period of each day. Light measurements were determined using a digital lux meter sensor.

### Secondary metabolite extraction and HPLC-ESI-MS/MS analysis

Leaf tissues for anthocyanin measurements were collected from nodes 4–6 from each plant at the end of the light period of each day to standardize any circadian or diurnal effects on the transcript abundance of anthocyanin biosynthetic genes. Pigments were extracted and analyzed with a previously reported method of HPLC-ESI-MS/MS^46^. Anthocyanin levels were determined as delphinidin-3-O-rutinoside equivalents by integrating peak areas. Similarly, kaempferol-3-O-rutinoside-7-O-glucoside levels were determined as kaempferol equivalents. HPLC separation was achieved using ACQUITY UPLC™ Xbridge BEH C18 columns (150 × 2.1 mm, 3.5 μm). Both the positive-ion (ESI^+^) and negative-ion (ESI^−^) modes were used in the MS system (Xevo G2-XS QTof, Waters Corp., Milford, MA, USA).

### Total anthocyanin analysis

The spectrophotometric differential pH method was applied for the measurement of total anthocyanins. Frozen samples (100 mg) were crushed into powder in liquid nitrogen and then extracted for further analysis^46^. The total anthocyanin contents of different samples were analyzed in triplicate.

### Measurement of chlorophyll

For the measurement of chlorophyll, a section of fruit pericarp was cut, weighed and ground into powder in a mortar with liquid nitrogen, and then total chlorophyll was extracted and examined with the method reported previously^47^.

### RNA extraction and quantitative real-time PCR analysis

Total RNA extraction and RT-qPCR were carried out as previously reported. Gene expression analysis in tomato plants was normalized to *SlCAC* as a reference gene^48^. Gene expression analysis in tobacco leaves was normalized to *NbActin* as a reference gene^49^. The values reported here represent three biological repeats for each sample.

### Plasmid construction and tomato transformation

The full-length open reading frame of SlAN2 cDNA from tomato was amplified using primers SlAN2F (5′-GCTCTAGAATGAATACTCCTATGTGTG-3′) and SlAN2R (5′-CGCAGAGCTCTTAATTAAGTAGATTCCATAA-3′). The amplification products were digested and inserted into the pBI121 plasmid to yield the overexpression plasmid Pro35S:SlAN2. The construction of the overexpression plasmid Pro35S:BrTT8 was described in a previous article^45^. In addition, the overexpression vector Pro35S:SlMYBL2 was generated by the same method with primers SlMYBL2F (5′-GCTCTAGAATGAGAAAGCCTTGTTGTG-3′) and SlMYBL2R (5′-CGCAGAGCTCCGAGAATGTCTTCGATACT-3′). Subsequently, all the final constructs were sequenced and introduced into wild-type tomatoes using the procedure described previously^45^. Stable transformed plants were selected on the basis of kanamycin resistance and genotyping.

### Yeast two-hybrid assay

To avoid self-activation, the carboxyl terminal-deleted forms of BrTT8 and SlTT8 (also named SlAN1 or AH) were excised by *EcoR*I/*Sal*I double digestion and subcloned into pGBKT7 to generate an in-frame fusion with the GAL4 DNA binding domain, respectively^32,33,41,42^. Full-length sequences of *SlAN2*, *SlMYBL2*, *SlAN11* and *BrTT8* were excised using *EcoR*I/*Xho*I double digestion and subcloned into pGADT7 to generate an in-frame fusion with the GAL4 activation domain. The yeast two-hybrid assays were performed using the Matchmaker^TM^ Gold Yeast Two-Hybrid System according to the manufacturer’s instructions (Clontech, Palo Alto, CA, USA).

### FRET measurements

The FRET efficiency between two fusion proteins, BrTT8-EGFP and SlAN2-mCherry, was measured in the epidermal cells of *Nicotiana benthamiana* cultivated in the greenhouse as described, and transient *Agrobacterium tumefaciens*-mediated gene expression was performed as described^50^.

### Transient assay

The open reading frames of BrTT8, SlAN2, SlMYBL2, EGFP, and mCherry were amplified with specific primers (Table S3) and cloned into pBI121 to obtain the expression vectors Pro35S:BrTT8-eGFP, Pro35S:SlAN2-mCherry, Pro35S:SlMYBL2-mCherry and Pro35S:SlMYBL2, respectively. Transient assays were conducted as previously reported^50^. Final measurements of secondary metabolites were performed four days after infiltration.

### Sequence analyses

A multiple sequence alignment was generated with DNAMAN version 5.2.2. A neighbor-joining tree was produced with 1000 bootstrap replicates using the program MEGA (Molecular Evolutionary Genetics Analysis) version 3.1.

### Statistical calculations

Unless otherwise indicated, all data were analyzed by one-way ANOVA, followed by Duncan’s multiple range test using the SPSS 17 program (SPSS 17.0, SPSS Inc., USA). Different letters indicate that the results were statistically significant at *P* < 0.05.

## Results

### Extensive anthocyanin accumulation in *Pro35S:BrTT8* tomato plants under natural high-light conditions

**Fig. 2:**
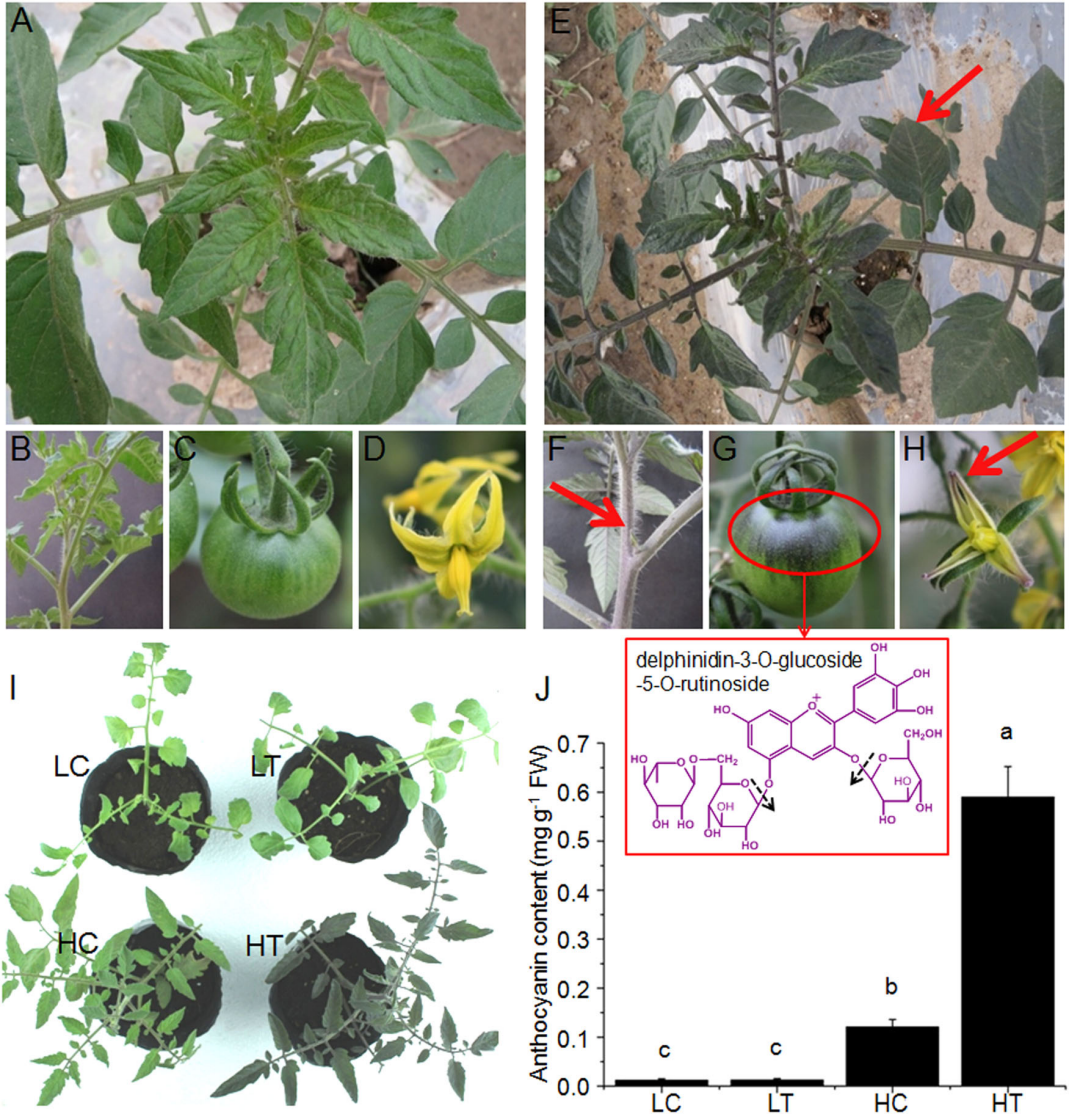
Anthocyanins accumulated widely in various tissues of *Pro35S:BrTT8* tomato plants grown under high-light conditions. Anthocyanins accumulated considerably in the foliage (**e**), stems (**f**), fruits (**g**) and flowers (**h**) of *Pro35S:BrTT8* plants, with wild-type (AC) plants grown under natural high-light conditions (700–2000 μmol photons m^−2^ s^−1^) as a control (**a−d**). The structure of the major anthocyanin, delphinidin-3-*O*-glucoside-5-*O*-rutinoside, identified in fruits is framed by a red box, and the major cleavage sites confirmed by mass spectrometry are indicated with dashed arrows. **i** Wild-type (AC) and *Pro35S:BrTT8* tomato seedlings grown in artificial low-light conditions (50–100 μmol photons m^−2^ s^−1^) and high-light conditions (700 μmol photons m^−2^ s^−1^) exhibit significant differences in anthocyanin accumulation. **j** Analysis of total anthocyanin contents in leaves of wild-type and *Pro35S:BrTT8* tomato seedlings under artificial low-light and high-light conditions. The individual plants shown are representative of the six plants grown per treatment. LC seedlings of control (AC) under low-light conditions, HC seedlings of control under high-light conditions, LT seedlings of transgenic (*Pro35S:BrTT8*) plants under low-light conditions, HT seedlings of transgenic plants under high-light conditions. Biological replicates were performed in triplicate, and different letters indicate a significant difference at *P* < 0.05

In wild-type tomato plants cultivated in natural high-light conditions (700–2000 μmol photons m^−2^ s^−1^), only a tiny amount of anthocyanins was observed in the epidermal cells of leaf veins. In contrast, anthocyanin pigmentation occurred widely in the leaves, stems, petals and fruits of *Pro35S:BrTT8* tomato plants (T0 generation) (Fig. 2a−h). Two types of glycosylated anthocyanins were separated and identified from the epicarps of *Pro35S:BrTT8* fruits (Supplemental Figure S1). Delphinidin-3-*O*-glucoside-5-*O*-rutinoside, which occupied 88% of the total amount of anthocyanins, is indicated by the red frame in Fig. 2 (Table 1). The structures and major cleavage sites of delphinidin-3-*O*-glucoside-5-*O*-rutinoside and delphinidin-3-*O*-glucoside-5-*O*-(*p*-coumaroyl)arabinoside, confirmed by mass spectrometry, are indicated with dashed arrows (Supplemental Figure S1). Apparently, delphinidin-based anthocyanins occupied the absolute number of percentages, indicating a specific biosynthetic pathway in tomato. Gene expression analysis indicates that BrTT8 promotes anthocyanin production probably by transcriptional activation of structural genes in leaves exposed to natural high light (Supplemental Figure S2).

**Table 1:** Anthocyanin contents (mg g^−1^ dry wt.) in the epicarps of wild-type and *Pro35S:BrTT8* fruits under natural high-light conditions (*n* = 3)

No.^a^	[M]^+^ (m/z)	MS/MS (m/z)	Anthocyanins	Low light		High light	
				WT^b^	Pro35S:BrTT8^c^	WT	Pro35S:BrTT8
1	773	465/303	Delphinidin-3-*O*-glucoside-5-*O*-rutinoside	Nd^d^	Nd	Nd	0. 53 ± 0. 08
2	743	465/303	Delphinidin-3-*O*-glucoside-5-*O*-(*p*-coumaroyl)arabinoside	Nd	Nd	Nd	0. 07 ± 0. 02
Total				Nd	Nd	Nd	0.60 ± 0.09

^a^No corresponds to the elution order in HPLC analysis (Supplemental Figure S1)

^b^WT indicates wild type

^c^Pro35S:BrTT8 indicates transgenic tomato plants with a high expression level of the heterologous gene *BrTT8* driven by the 35S promoter

^d^Nd indicates that no anthocyanin was detected

Compared with natural high-light conditions, *Pro35S:BrTT8* tomato plants exhibited an anthocyaninless phenotype under low-light conditions (50–100 μmol photons m^−2^ s^−1^) (Fig. 2i). To investigate the effects of light intensity on anthocyanin pigmentation in tomato plants, seedlings of wild-type and *Pro35S:BrTT8* (T1 generation) plants were grown under low-light conditions and then treated with artificial high light (700 μmol photons m^−2^ s^−1^). Compared with anthocyaninless seedlings of wild-type and transgenic tomato plants under low-light conditions, anthocyanin production was enhanced significantly in seedlings under high-light conditions, especially in *Pro35S:BrTT8* tomato plants (Fig. 2i, j). In particular, the leaves of *Pro35S:BrTT8* tomato plants exposed to high light contained a high concentration of anthocyanins (0.56 mg g^−1^ FW), while wild-type seedlings under the same conditions displayed a much lower intensity of anthocyanin pigmentation (<0.13 mg g^−1^ FW) (Fig. 2j).

### Transcript changes of anthocyanin biosynthetic and regulatory genes in *Pro35S:BrTT8* seedlings under different light conditions

**Fig. 3:**
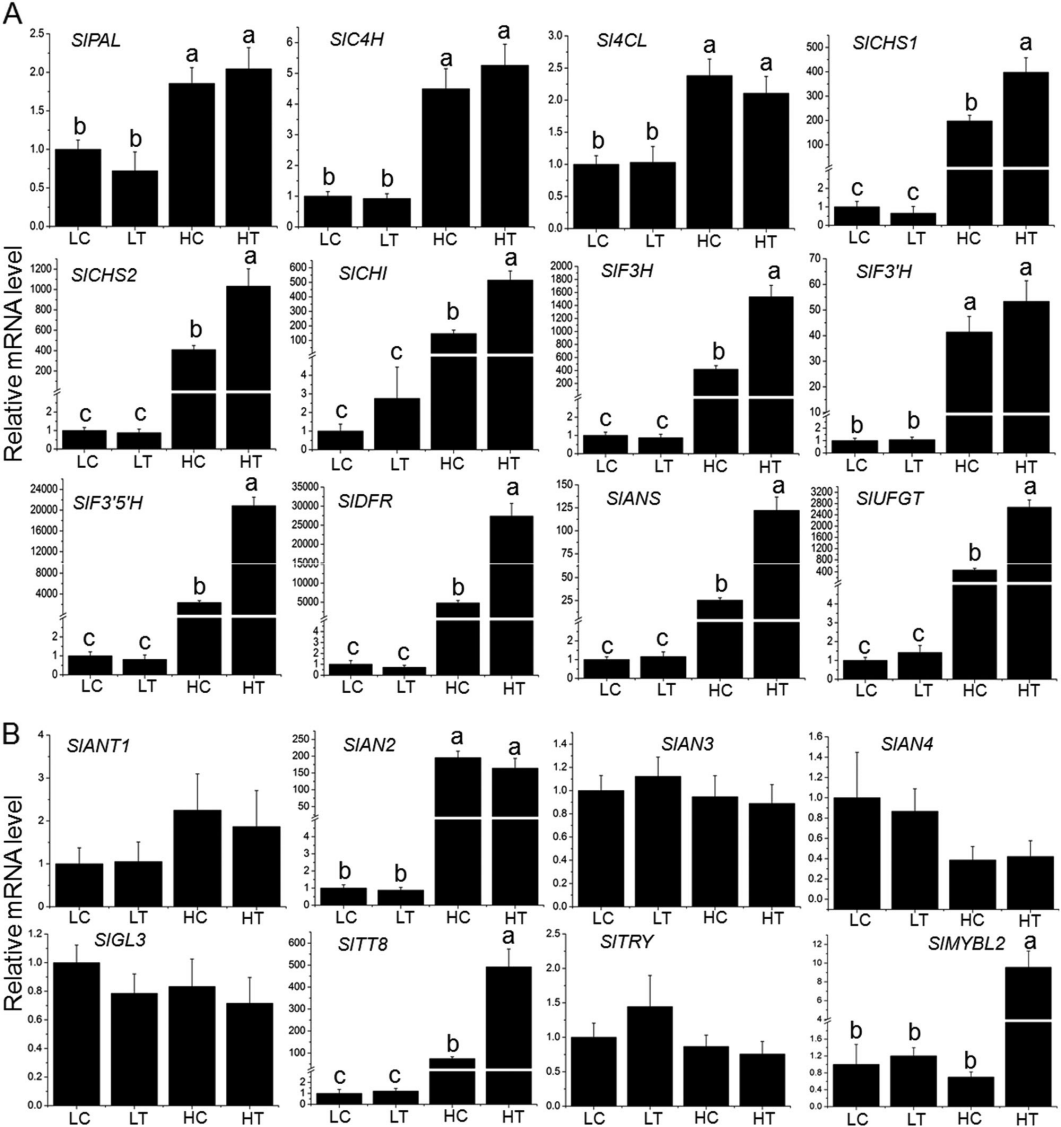
Expression analysis of the anthocyanin biosynthesis related genes in wild-type and transgenic leaves under different light conditions. Expression analysis of the anthocyanin biosynthetic genes (**a**) and key regulatory proteins (**b**) in leaves of wild-type and *Pro35S:BrTT8* tomato seedlings grown under artificial high-light and low-light conditions. The longitudinal axis indicates the expression levels of genes relative to the level of *SlCAC*. Biological replicates were performed in triplicate, and different letters indicate a significant difference at *P* < 0.05

The expression levels of almost all anthocyanin biosynthetic genes were clearly increased in both transgenic and wild-type plants (HT and HC) under artificial high-light conditions compared with plants under low-light conditions (LT and LC) (Fig. 3a). As reported before, the increase of gene transcripts is often congruously paralleled by respective protein changes^16^, which then lead to large amounts of metabolite production. Anthocyanin biosynthetic genes, including *PAL*, *C4H*, *4CL*, *CHS1*, *CHS2*, *CHI*, *F3H*, *F3′H*, *F3′5′H*, *DFR*, *ANS* and *UFGT*, showed high transcript abundance in wild-type and *Pro35S:BrTT8* seedlings under high-light conditions and very weak signals for *F3′H*, *F3′5′H*, *DFR* and *UFGT* under low-light conditions. These results indicate that the expression of most structural genes was dramatically induced by high light. Specifically, F3′5′H and DFR exhibited the highest fold increases in transgenic plants under high-light exposure (Fig. 3a). On the whole, transgenic plants showed significantly enhanced expression for most of the biosynthetic genes, including *CHS1*, *CHS2*, *CHI*, *F3H*, *F3′H*, *F3′5′H*, *DFR*, *ANS* and *UFGT*, compared with wild-type plants under high-light conditions. However, the expression levels of *PAL*, *C4H* and *4CL*, which encode enzymes catalyzing the first three steps of the anthocyanin biosynthetic pathway, did not show obvious differences between wild-type and transgenic plants under high-light conditions. In addition, *BrTT8* transcripts were shown to be constantly expressed under both high-light and low-light conditions (Supplemental Figure S3).

All the regulatory genes examined were expressed at similar background levels in *Pro35S:BrTT8* and wild-type plants under low-light conditions, and the transcripts of most transcription factors remained relatively constant among samples of HT, HC, LT and LC (Fig. 3b). Nonetheless, the expression of *SlAN2* and *SlTT8* was enhanced significantly by high light (Fig. 3b). Furthermore, the gene expression of *SlTT8* in *Pro35S:BrTT8* plants was evidently higher than that of wild-type plants, indicating that BrTT8 further enhanced the expression of SlTT8 under high-light treatment. Unexpectedly, the expression of *SlMYBL2*, a putative transcription repressor, was also upregulated significantly in *Pro35S:BrTT8* plants under artificial high light.

### Dynamics of anthocyanin accumulation and associated gene expression in *Pro35S:BrTT8* seedlings under high-light treatments

**Fig. 4:**
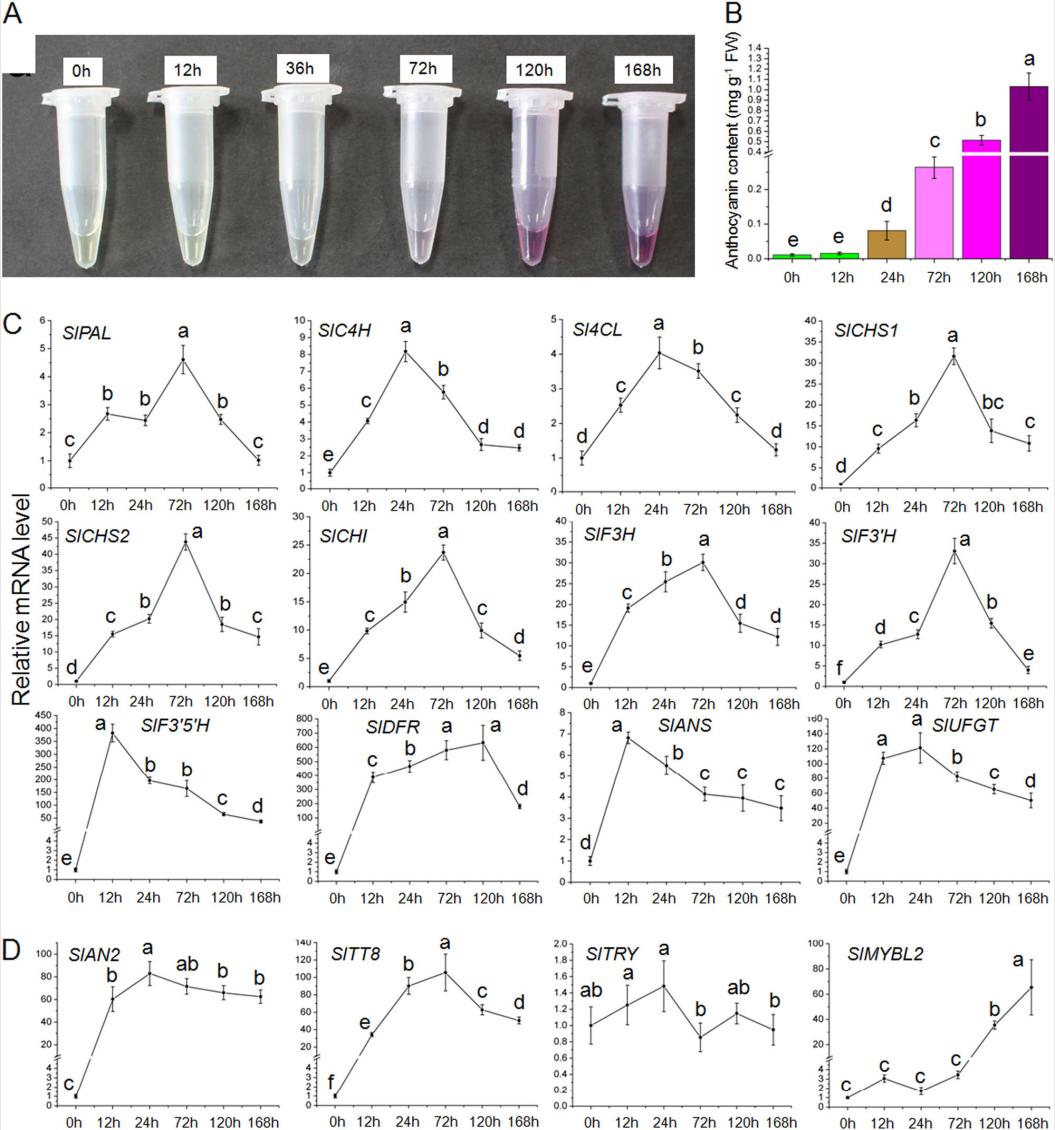
Dynamics of anthocyanin accumulation and gene expression in *Pro35S:BrTT8* tomato seedlings under artificial high-light conditions. Preliminary pigment extraction of transgenic seedlings treated with high light for different time periods (**a**) and analysis of total anthocyanin contents (**b**). Transcripts of anthocyanin biosynthetic genes (**c**) and key regulatory genes (**d**) in leaves of transgenic tomato seedlings treated with high light were analyzed at various stages. The longitudinal axis indicates the gene expression levels relative to *SlCAC*. Biological replicates were performed in triplicate, and different letters indicate a significant difference at *P* < 0.05

The simultaneous enhanced expression of the putative transcriptional activators SlAN2 and SlTT8 and the repressor SlMYBL2 make it difficult to explain the underlying mechanisms of the profound production of anthocyanins in *Pro35S:BrTT8* seedlings under high-light exposure. To obtain a better sense of how transcription factors coordinate the regulation of anthocyanin biosynthesis, the dynamics of anthocyanin accumulation and associated gene expression were analyzed. *Pro35S:BrTT8* seedlings cultivated in low-light conditions were transferred to artificial high-light conditions for 7 days. Leaves were sampled from transgenic seedlings at consistent times after 0, 12, 36, 72, 120 and 168 h, and then total anthocyanin contents and the expression of anthocyanin biosynthetic and regulatory genes were examined. First, transcripts of *BrTT8* were found to be stable in transgenic seedlings at different stages (Supplemental Figure 4). As shown in Fig. 4, anthocyanin production was absent in samples gathered at 0 and 12 h, while it rose significantly at 36 h and reached its maximum value at 168 h. Transcripts of all the structural genes increased swiftly just after the onset of high-light treatment and reached peak values within 72 h. Thereafter, transcripts of all the structural genes except *SlPAL*, *SlC4H* and *Sl4CL* gradually declined to relatively stable levels, which were still drastically higher than the respective initial levels at 0 h (Fig. 4). These results show that anthocyanin accumulation lagged behind the transcriptional activation of structural genes in tomato seedlings upon high-light treatment.

As shown in Fig. 4, the transcripts of *SlAN2* and *SlTT8* increased drastically just after the onset of high-light treatment. In particular, transcripts of *SlAN2* increased to their maximal values within 12 h and remained relatively stable at the remaining time points. However, transcripts of *SlTT8* increased slowly to their peak value and then displayed a declining trend. The dynamics of *SlTT8* transcripts resembled those of some anthocyanin structural genes mentioned above, suggesting that *SlTT8* might be transcriptionally regulated in a similar manner at high-light exposure. However, the transcripts of *SlMYBL2* remained relatively stable within the first 72 h but increased substantially near the end of the high-light treatment. Apparently, SlMYBL2 did not participate in the transcriptional regulation of anthocyanin structural genes at the earlier stage of the high-light treatment. In addition, the expression of the putative transcriptional repressor SlTRY, belonging to R3-MYB family, seemed insensitive to high-light stress.

### Nonuniform anthocyanin pigmentation in fruits of *Pro35S:BrTT8* plants after exposure of natural high light

**Fig. 5:**
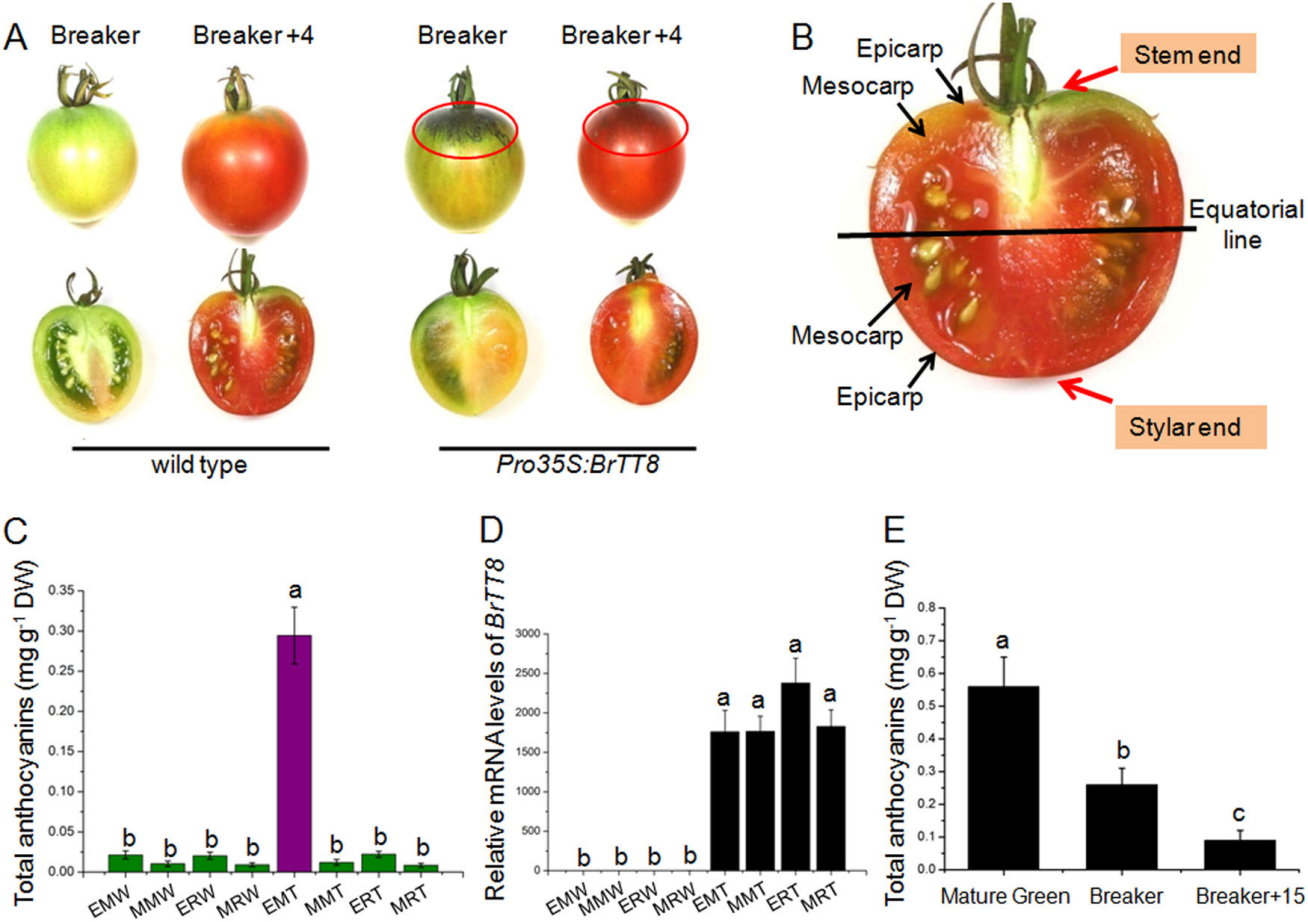
Anthocyanins accumulated specifically in the upper epicarps of *Pro35S:BrTT8* tomato plants grown under natural high-light conditions. **a** Regardless of the developmental stages, anthocyanins accumulated specifically in the upper epicarps of fruits under natural high-light conditions. **b** Schematic representation of the different parts of fruit used for pigment measurements and gene expression analysis. **c** Total anthocyanin contents in different parts of wild-type and *Pro35S:BrTT8* tomato fruits at the Breaker + stage. **d** Expression analysis of *BrTT8* in different fruit parts of wild-type and *Pro35S:BrTT8* plants. The longitudinal axis indicates the expression level of *BrTT8* relative to *SlCAC*. **e** Total anthocyanin contents of epicarps in the stem end in *Pro35S:BrTT8* tomato fruits at different development stages. Breaker + 4 4 days after breaker, Breaker + 15 15 days after breaker, EMW epicarp at the stem end of wild-type fruits, MMW mesocarp at the stem end of wild-type fruits, ERW epicarp at the stylar end of wild-type fruits, MRW mesocarp at the stylar end of wild-type fruits, EMT epicarp at the stem end of transgenic fruits, MMT mesocarp at the stem end of transgenic fruits, ERT epicarp at the stylar end of transgenic fruits, MRT mesocarp at the stylar end of transgenic fruits. Biological replicates were performed in triplicate, and different lowercase letters indicate significance at *P* < 0.05

Visual inspection showed that anthocyanins accumulated specifically in roughly spherical areas of the upper epicarp around the stem end of *Pro35S:BrTT8* fruits under natural high light, regardless of developmental stage (Fig. 5a, c). In addition, the total anthocyanin content of epicarps at the stem end of ripening fruits at the breaker stage was 0.26 mg g^−1^ dry weight, which was evidently lower than that of fruits at the green stage, suggesting that anthocyanins degrade notably during the fruit ripening process (Fig. 5e). However, no anthocyanins were detected in any fruit tissues of wild-type tomato plants under natural high-light conditions.

**Fig. 6:**
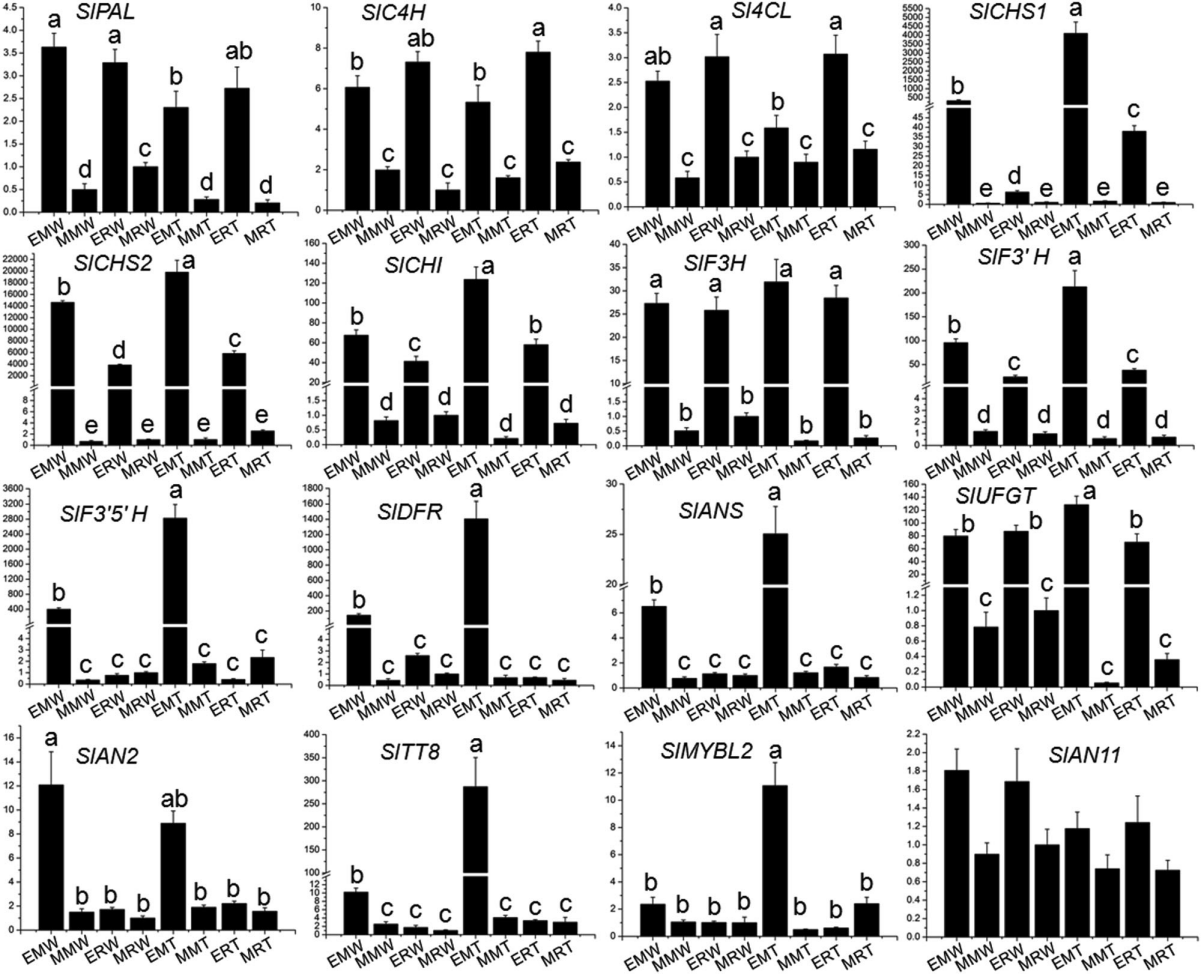
Expression profiles of anthocyanin biosynthetic and regulatory genes in different fruit parts of wild-type and *Pro35S:BrTT8* tomato plants under natural high-light conditions. The longitudinal axis indicates the expression levels of genes relative to *SlCAC*. Biological replicates were performed in triplicate, and different lowercase letters indicate significance at *P* < 0.05

To investigate the molecular mechanisms underlying the region-specific anthocyanin pigmentation in tomato fruits of *Pro35S:BrTT8* plants under natural high-light conditions, expression levels of the associated genes were analyzed in divided parts of wild-type and transgenic fruits as follows: EMW (epicarp at stem end of wild-type plants), MMW (mesocarp at stem end of wild-type plants), ERW (epicarp at stylar end of wild-type plants), MRW (mesocarp at stylar end of wild-type plants), EMT (epicarp at stem end of transgenic plants), MMT (mesocarp at stem end of transgenic plants), ERT (epicarp at stylar end of transgenic plants) and MRT (mesocarp at stylar end of transgenic plants) (Fig. 5b). Before this, *BrTT8* transcripts were examined preferably and were consistently expressed in different regions of *Pro35S:BrTT8* fruits (Fig. 5d). In fruits of wild-type plants, the expression levels of SlCHS1, SlCHS2, SlCHI, SlF3′H, SlF3′5′H, SlDFR and SlANS in epicarps at the stem end were significantly higher than those at the stylar end. Moreover, the expression levels of these genes were further enhanced on a large scale in epicarps at the stem end of *Pro35S:BrTT8* fruits. However, only weak transcriptional signals for *SlCHS1*, *SlCHS2*, *SlCHI*, *SlF3H*, *SlF3′H*, *SlF3′5′H*, *SlDFR*, *SlANS* and *SlUFGT* were detected in mesocarps of wild-type plants at both the stem end and stylar end. In addition, these genes exhibited no evident differences in mesocarps of wild-type and *Pro35S:BrTT8* plants (Fig. 6). These results provide a good explanation for the large amounts of flavonoid accumulation that occur in epicarps but not in mesocarps^40^.

In the wild-type epicarps, the transcripts of *SlAN2* and *SlTT8* in EMW were significantly higher than those in MMW, ERW, and MRW (Fig. 6). Similar expression patterns for *SlAN2* and *SlTT8* were also found in *Pro35S:BrTT8* fruits. Moreover, the expression levels of *SlTT8* and *SlMYBL2* were further enhanced in EMT compared with EMW. Among the regulatory genes that showed increased transcripts in EMT, *SlTT8* was strongly upregulated (more than 20-fold) in comparison with EMW. Altogether, these results suggest that the region-specific expression of SlAN2, SlTT8 and SlMYBL2 should be responsible for the unique pigmentation pattern in fruits of *Pro35S:BrTT8* plants exposed to natural high light.

### BrTT8 physically interacts with SlAN2, SlAN11, and SlMYBL2

**Fig. 7:**
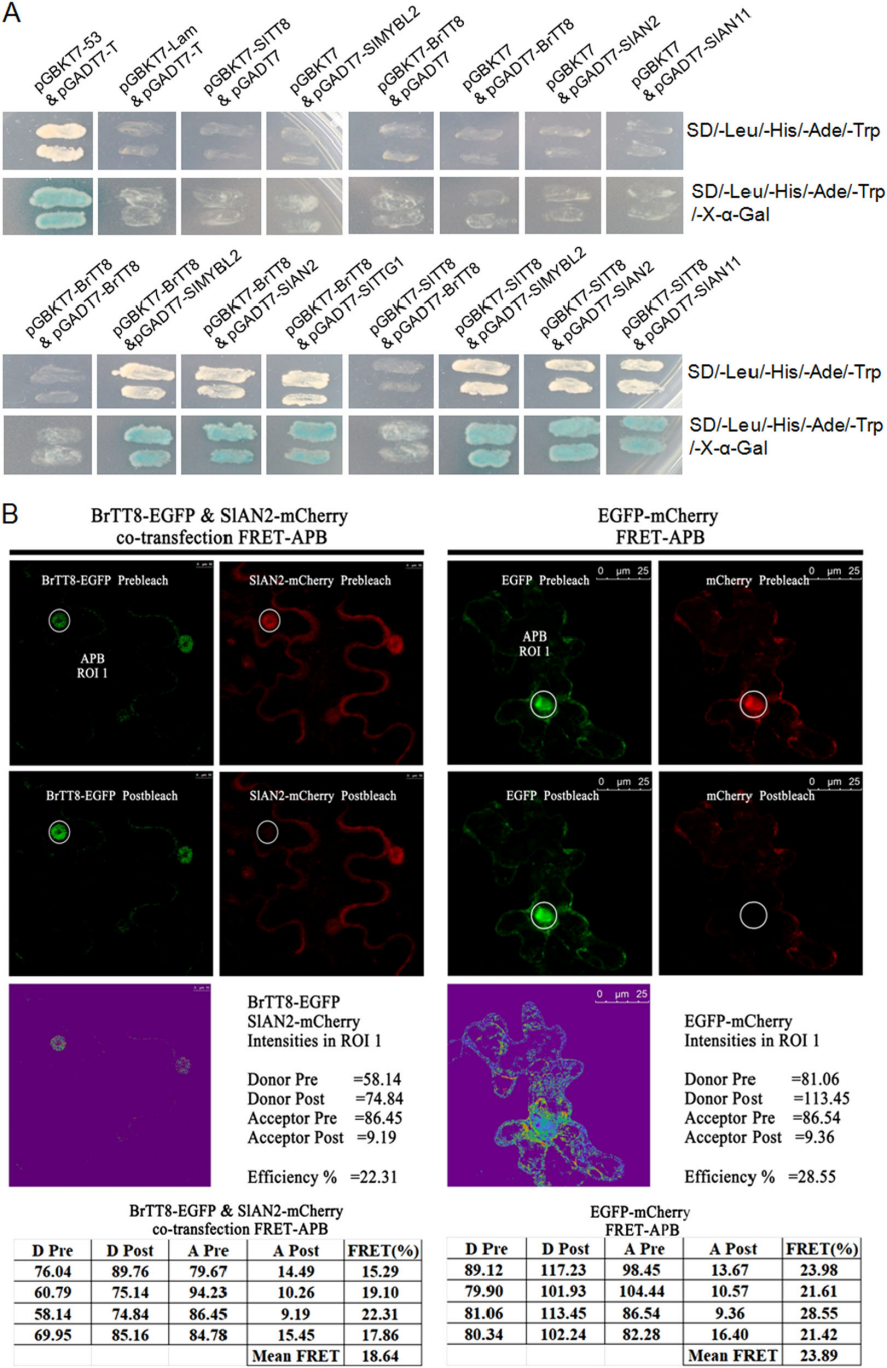
BrTT8 interacts physically with SlAN2, SlAN11, and SlMYBL2 in vivo. **a** Protein−protein interactions between members of the MBW regulatory complex were analyzed by yeast two-hybrid assays. **b** Physical interactions between BrTT8 and SlAN2 were further confirmed by fluorescence resonance energy transfer (FRET)

Multiple sequence alignment of BrTT8, SlTT8 and other bHLH anthocyanin regulators suggests that all the proteins share a typical bHLH domain and a Myb interaction region at the N terminus (Supplemental Figure S5). In addition, phylogenetic analysis of bHLH proteins from various plant species indicates that SlTT8 is an ortholog of BrTT8, a functional anthocyanin activator (Supplemental Figure S6). Similarly, sequence analysis of MYB proteins from various species showed that SlAN2 and SlMYBL2 share the same conserved bHLH protein interaction motif with other transcriptional regulators (Supplemental Figures S7 and S8). To further unravel the molecular mechanism by which high light regulates anthocyanin accumulation in the leaves and fruits of *Pro35S:BrTT8* plants, Y2H assays were performed to identify candidate proteins that physically interact with BrTT8. Positive X-a-Gal activity was found in strains containing pGBKT7-BrTT8 & pGADT7-SlAN2, pGBKT7-BrTT8 & pGADT7-SlMYBL2 and pGBKT7-BrTT8 & pGADT7-SlAN11 grown on the QDO/X-a-Gal screening medium but not in those containing pGBKT7-BrTT8 & pGADT7-BrTT8 (Fig. 7a), indicating that BrTT8 interacts with SlAN2, SlMYBL2 and SlAN11 physically. An MYB-bHLH-WD40 protein complex that plays an essential regulatory role in anthocyanin biosynthesis has been studied extensively in previous reports^21,51^. In addition, large amounts of transcripts of *SlAN2*, *BrTT8*, *SlMYBL2* and *SlAN11* were detected in tissues exhibiting abundant anthocyanin accumulation. Consequently, it is reasonable to speculate that *Pro35S:BrTT8* plants adapt to high-light conditions by enhancing anthocyanin pigmentation in vegetative and fruit tissues via the transcriptional activity of a heterogeneous MBW complex consisting of BrTT8, SlAN2 and SlAN11. Additionally, the physical interaction between SlMYBL2 and BrTT8 indicates that SlMYBL2 might inhibit the assembly of the functional MYB-bHLH-WD40 protein complex by competitively binding with the MYB interaction domain in BrTT8. Positive X-a-Gal activity was also found in yeast strains containing pGBKT7-SlTT8 & pGADT7-SlAN2, pGBKT7-SlTT8 & pGADT7-SlMYBL2 and pGBKT7-SlTT8 & pGADT7-SlAN11 grown on QDO/X-a-Gal screening medium but not in those containing pGBKT7-SlTT8 & pGADT7-BrTT8 (Fig. 7a), suggesting that SlTT8 could interact with the endogenous anthocyanin regulators physically but not with BrTT8. These results indicate that SlTT8 might also participate in the assemblage of the endogenous MBW complex with SlAN2 and SlAN11. Thus, the MBW complex consisting of endogenous transcription factors SlTT8, SlAN2 and SlAN11 might also contribute to anthocyanin accumulation. It seems that BrTT8 cannot form a homodimer with itself or a heterodimer with SlTT8 in vivo. However, it is worth noting that the C-terminal regions of the *BrTT8* and *SlTT8* genes were deleted in Y2H assays for the elimination of potential transcriptional activation activity.

To confirm the physical interaction between BrTT8 and SlAN2, the full-length coding sequences of BrTT8 and SlAN2 were fused with EGFP and mCherry protein, respectively. BrTT8 interacted with SlAN2 in the FRET-APB assays (Fig. 7b), confirming the presence of a heterogeneous MBW complex in *Pro35S:BrTT8* tomato plants. Additionally, both BrTT8 and SlAN2 were located in the nucleus of agroinfiltrated tobacco epidermal cells visualized with laser scanning confocal microscopy. Altogether, these results support the hypothesis that a heterogeneous MBW complex consisting of BrTT8, SlAN2 and SlAN11 fundamentally regulates anthocyanin accumulation.

### BrTT8, SlAN2 and SlMYBL2 coordinately regulate the biosynthesis of anthocyanins and polyphenols in tobacco

**Fig. 8:**
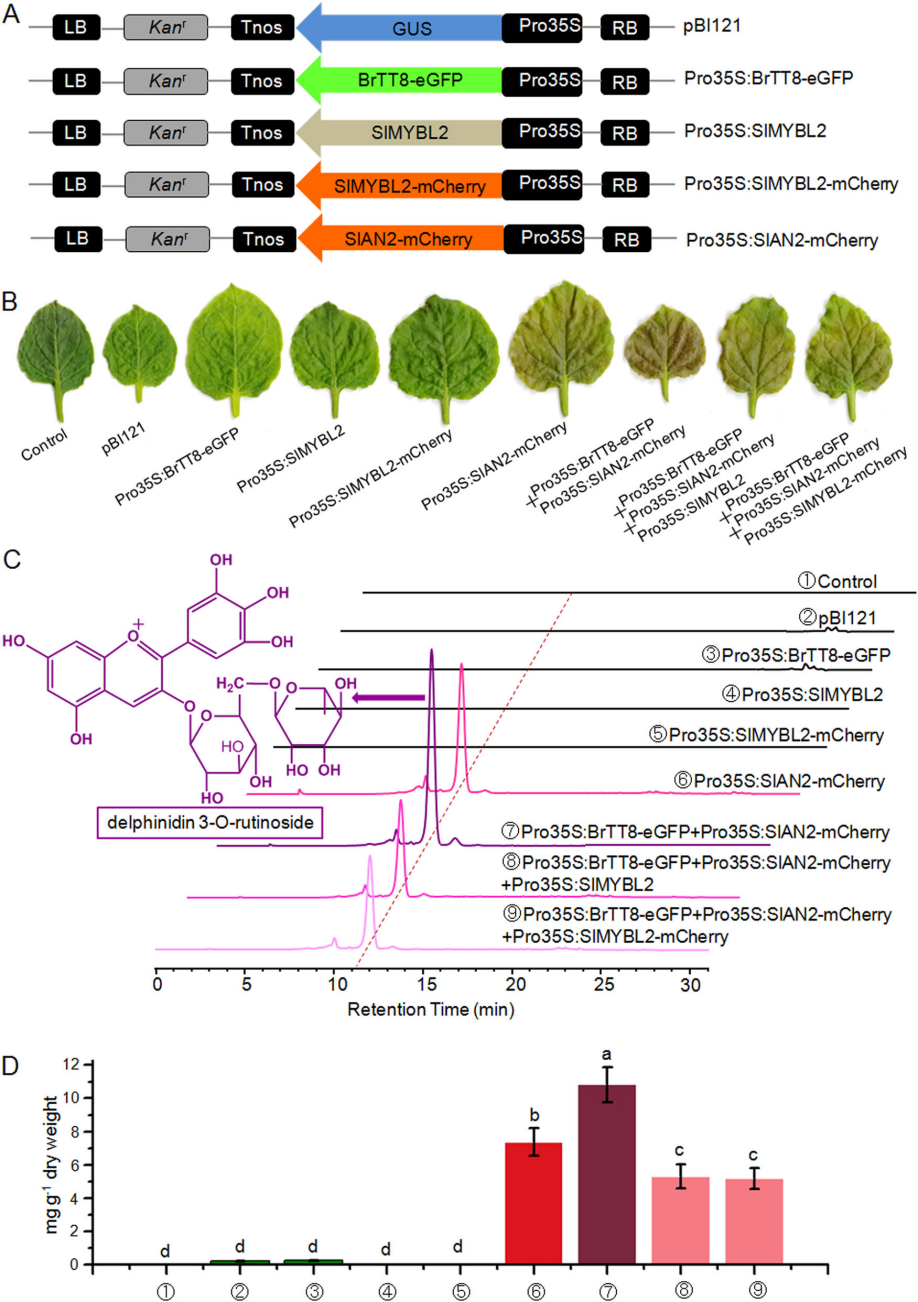
SlAN2, BrTT8, and SlMYBL2 coordinately regulated the biosynthesis of anthocyanins in tobacco leaves. **a** Schematic representation of the overexpression constructs used for transformation of *Nicotiana benthamiana* leaves. **b** Anthocyanin pigmentation in *Nicotiana benthamiana* leaves 4 days after ectopic expression of different combinations of exogenous genes. **c** HPLC profiles of anthocyanins extracted from infiltrated patches 4 days after infiltration, and the structure of the main anthocyanin component identified by HPLC-ESI-MS/MS is shown in purple color. **d** Total anthocyanin contents of infiltrated patches in the control and leaves expressing different combinations of exogenous genes. Biological replicates were performed in triplicate, and different lowercase letters indicate significance at *P* < 0.05

To further verify the hypothesis proposed above, expression vectors were generated following the schematic representation shown in Fig. 8a and then used for transient transformation of tobacco epidermal cells. Four days after infiltration, purple pigments emerged clearly in the epidermal tissues of tobacco leaves (Fig. 8b). Similar to the control, overexpression of SlMYBL2 did not result in the accumulation of purple pigments. Moreover, sole expression of BrTT8 failed to induce anthocyanin accumulation in infiltrated leaves, indicating inefficient interactions with the endogenous MYB and WD40 proteins or expression deficiency of endogenous MYB and WD40 proteins. In contrast, the ability of SlAN2 to induce anthocyanin accumulation, without coinfiltration of a bHLH protein, might be due to efficient interactions with the endogenous bHLH proteins in tobacco.

To further investigate the physical interactions between subunits of the MBW complex, different combinations of overexpression constructs were introduced into tobacco leaves by *Agrobacterium tumefaciens*-mediated transient expression. Among the infiltrated leaves, coinfiltration of BrTT8 and SlAN2 induced the highest amount of anthocyanin production; infiltration of SlAN2 induced the second highest; coinfiltration of BrTT8, SlAN2 and SlMYBL2 induced the third highest (Fig. 8b, d). In addition, delphinidin-3-*O*-runtinoside, as the main component of purple pigments, was extracted from all infiltrated patches and identified by HPLC-ESI-MS/MS (Fig. 8c), indicating that biosynthetic genes of the anthocyanin pathway were upregulated by the same mechanisms. Then, the transcript levels of anthocyanin biosynthetic and exogenous genes were examined in all the infiltrated patches (Supplemental Table S4 and Figure S9). Clearly, SlAN2, BrTT8 and endogenous WD40 protein coordinately promote anthocyanin pigmentation by activating the transcription of anthocyanin structural genes in tobacco cells, while SlMYBL2 prevents this transcriptional activation, probably by disturbing the formation of a functional MBW protein complex. Taking all these results into account, we can obtain three conclusions: (1) a functional MBW complex consisting of BrTT8, SlAN2, and endogenous WD40 protein is assembled efficiently in infiltrated tobacco leaves; (2) SlMYBL2 blocks high amounts of anthocyanin accumulation by preventing the assemblage of the functional MBW complex; and (3) the inability of BrTT8 to induce anthocyanin accumulation should be due to a deficiency in the expression of endogenous MYB proteins in tobacco.

Except for anthocyanins, the increased contents of phenolic acids and flavonols were also analyzed in infiltrated tobacco leaves (Table 2). The increased biosynthesis of neochlorogenic acid, chlorogenic acid, and cryptochlorogenic acid might be due to the upregulation of early anthocyanin biosynthetic genes, including *NbPAL* and *NbC4H*, since the biosynthesis of anthocyanin and phenolic acids share the same pathway in the initial steps of phenylpropanoid metabolism (Fig. 1). Similarly, it is possible that the enhanced biosynthesis of dihydroflavonol in the anthocyanin pathway contributes to the accumulation of kaempferol-3-O-rutinoside-7-O-glucoside (Table 2).

**Table 2:** Compound levels (mg g^−1^ dry wt.) of the tobacco leaves infiltrated by different *Agrobacterium tumefaciens* strains (*n* = 3)

No.	[M-H]^−^ (m/z)	MS/MS (m/z)	Polyphenols	Tobacco leaves infected by different expression vectors								
				①	②	③	④	⑤	⑥	⑦	⑧	⑨
1	353.0899	191.0570	Neochlorogenic acid	0.38 ± 0.04 ^cd^	0.47 ± 0.06^c^	0.61 ± 0.05^b^	0.34 ± 0.04^d^	0.42 ± 0.05^c^	0.61 ± 0.07^b^	0.61 ± 0.08^b^	0.75 ± 0.0^a^	0.74 ± 0.08^a^
2	353.0869	191.0579	Chlorogenic acid	1.17 ± 0.12^e^	1.63 ± 0.15^c^	1.68 ± 0.20^c^	1.33 ± 0.18^d^	1.67 ± 0.15^c^	2.68 ± 0.32^b^	3.81 ± 0.52^a^	3.28 ± 0.37^ab^	3.29 ± 0.42^ab^
3	353.0880	191.0559	Cryptochlorogenic acid	0.62 ± 0.10^c^	0.89 ± 0.07^b^	0.96 ± 0.05^b^	0.76 ± 0.06^c^	0.88 ± 0.11^b^	0.89 ± 0.12^b^	1.77 ± 0.21^a^	1.56 ± 0.17^a^	1.55 ± 0.11^a^
4	755.2028	593.506/285.0394	Kaempferol-3-*O*-rutinoside-7-*O*-glucoside	0.08 ± 0.02^c^	0.11 ± 0.02^b^	0.07 ± 0.02^c^	0.07 ± 0.02^c^	0.11 ± 0.04^b^	0.11 ± 0.03^b^	0.22 ± 0.05^a^	0.21 ± 0.03^a^	0.22 ± 0.03^a^
Total				2.25 ± 0.16	3.14 ± 0.18	3.42 ± 0.23	2.66 ± 0.21	3.09 ± 0.20	4.29 ± 0.35	6.41 ± 0.59	5.80 ± 0.43	5.81 ± 0.49

①, ②, ③, ④, ⑤, ⑥, ⑦, ⑧ and ⑨ reference the samples analyzed in Fig. 8c. Biological replicates were performed in triplicate, and different lowercase letters indicate significance at *P* < 0.05

### BrTT8, SlAN2, and SlAN11 coordinately trigger anthocyanin production in tomato plants

**Fig. 9:**
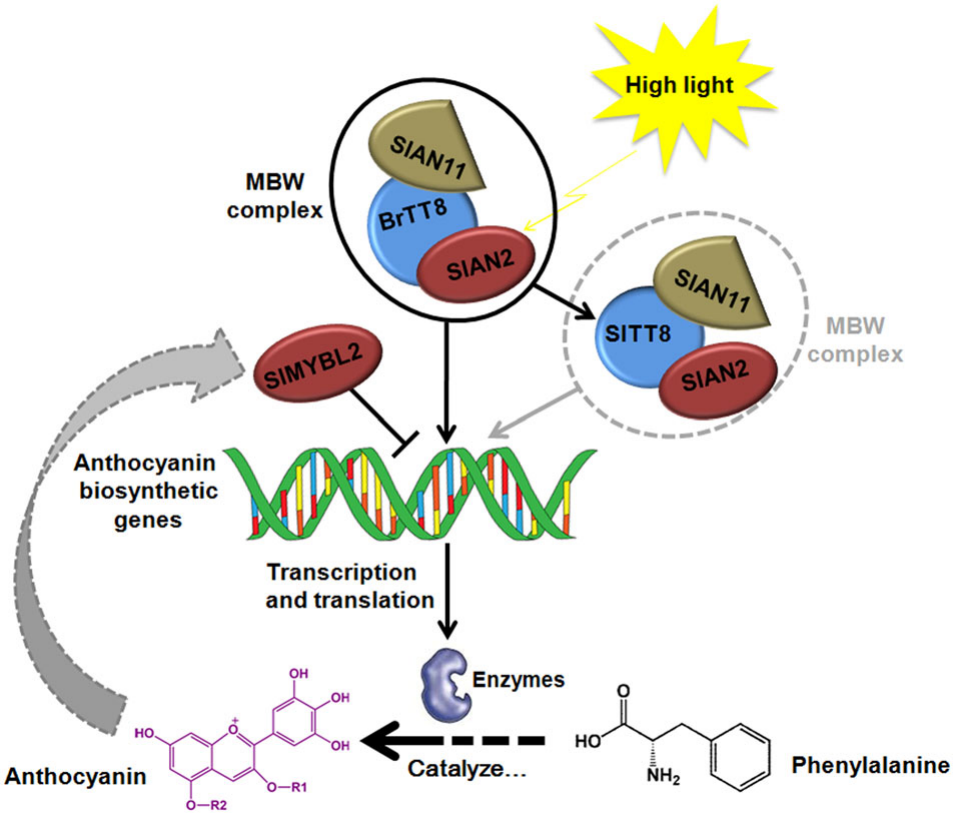
A proposed model summarizing that a heterogeneous MBW protein complex consisting of BrTT8, SlAN2, and SlAN11 triggers anthocyanin accumulation by increasing transcripts of the biosynthetic genes in tomato plants grown under high-light conditions

Together, a working model was proposed in which a heterogeneous MBW protein complex consisting of BrTT8, SlAN2, and SlAN11 triggers anthocyanin accumulation by increasing transcripts of biosynthetic genes in *Pro35S:BrTT8* tomato plants grown under high-light conditions (Fig. 9). Furthermore, an endogenous MBW protein complex consisting of SlTT8, SlAN2, and SlAN11 might also contribute to the transcriptional activation of anthocyanin biosynthesis in wild-type plants and enhance anthocyanin pigmentation further in *Pro35S:BrTT8* tomato plants under high-light conditions. However, large amounts of anthocyanin accumulation in plant tissues certainly consumes considerable amounts of precursor phenylalanine and energy, leading to a clear growth delay to some extent^52^. Consequently, too much anthocyanin pigmentation might activate the expression of SlMYBL2 through unknown mechanisms to avoid excess anthocyanin accumulation in a negative-feedback manner.

### **N**onuniform anthocyanin accumulation and gradient light flux received by epicarp cells in *Pro35S:BrTT8* plants

Fruits with uniform pigmentation always show better presentation and some tomato cultivars truly display uneven distribution of pigments in fruits^53^. The region-specific anthocyanin pigmentation makes *Pro35S:BrTT8* tomato a good model for metabolic and molecular studies of nonuniform fruit pigmentation in the plant kingdom. It was proven that light intensity has significant effects on anthocyanin accumulation in tomato plants in the earlier part of this article. As a result, the relationship between anthocyanin pigmentation patterns and direct high-light irradiance shed on epicarps of *Pro35S:BrTT8* tomato plants was investigated.

**Fig. 10:**
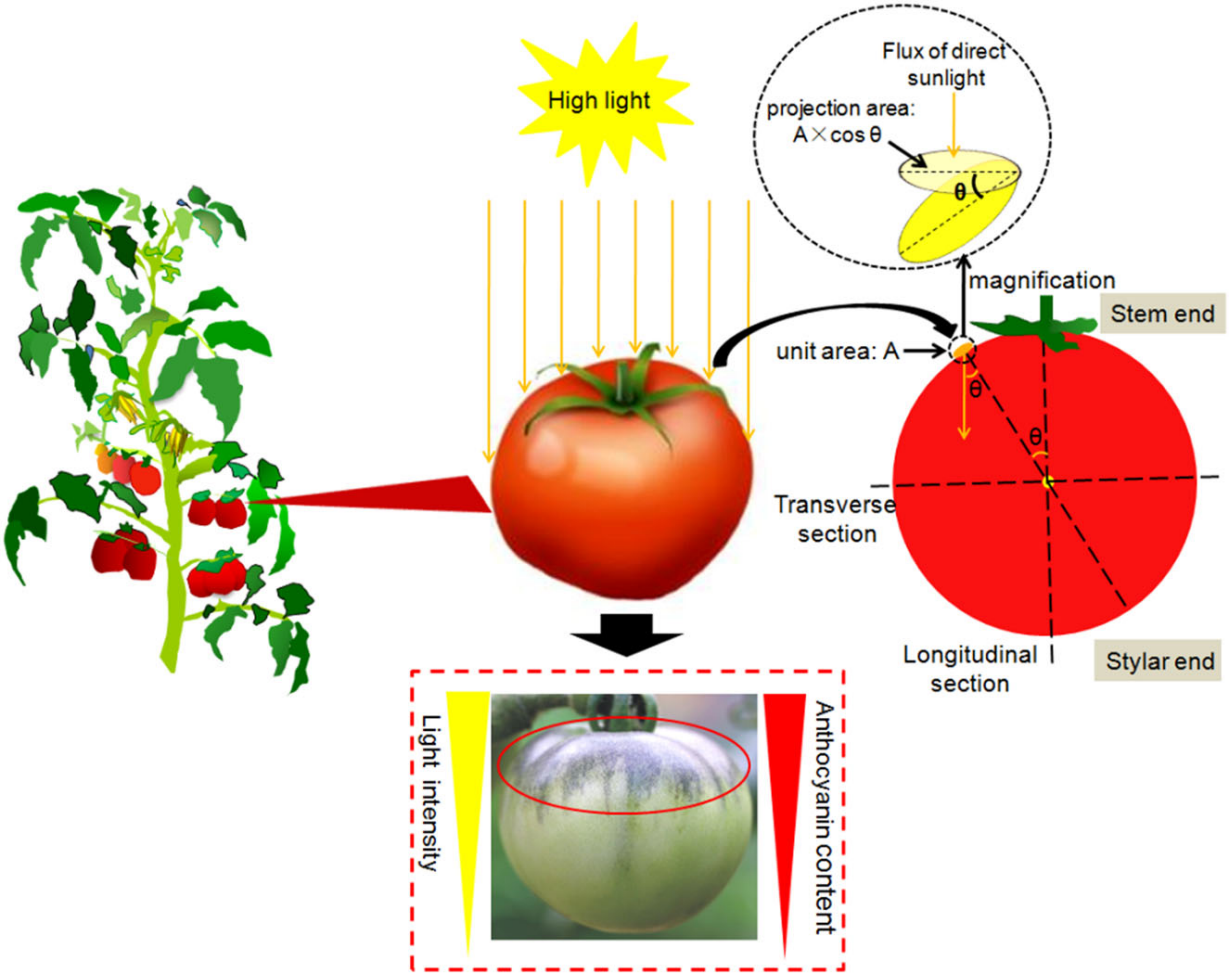
Schematic representation of nonuniform anthocyanin distribution and direct sunlight received by epicarp tissues of *Pro35S:BrTT8* tomato fruits under natural high-light conditions

As shown in Fig. 10, plant cells of mesocarps do not have the opportunity to absorb direct sunlight, and epicarps of the stylar end can only absorb scattered light (lower than 100 μmol photons m^−2^ s^−1^), which is much lower than direct sunlight in light intensity. In fact, the actual direct light flux received by the epidermal cells in the unit area changes as the position of the spherical fruit surface moves (Fig. 10). As the upper surface of the tomato fruit is an approximate regular sphere, it is easy to estimate that the actual luminous flux received per unit area on the fruit epicarp was only equal to its projection area in the horizontal surface. Supposing that the value of luminous flux passing through a unit area at a unit time is A under natural high light, the value of luminous flux received in the same unit area of the epicarp was A multiplied by cosine *θ*, where *θ* indicates the angle between a vertical line and a ligature connecting the unit area and sphere center (Fig. 10). Apparently, with the increase of the angle index, the value of cosine *θ* gradually decreases to zero, indicating a continuous reduction in the luminous flux of direct sunlight received in the unit area on the fruit surface. Consequently, the uneven flux of direct sunlight received by epidermal tissues provides a potential explanation for nonuniform anthocyanin pigmentation in tomato fruits upon exposure to natural sunlight. Moreover, the changing intensity of anthocyanin pigmentation in the upper epidermis is closely associated with the specific angle *θ*, determined by a fixed position. No visible purple pigments were observed in the upper epidermal tissues near the equatorial line of fruit because the direct sunlight flux received by the corresponding tissues was very limited. These results indicated that the activation of anthocyanin biosynthesis requires a certain strength of direct high-light irradiance. In other words, once the direct high-light flux received by the cells on the epicarp is less than the minimum limit, the cells are unable to trigger anthocyanin biosynthesis.

### Low-light irradiance cannot trigger anthocyanin accumulation in the upper epicarp of *Pro35S:BrTT8* tomato fruits

**Fig. 11:**
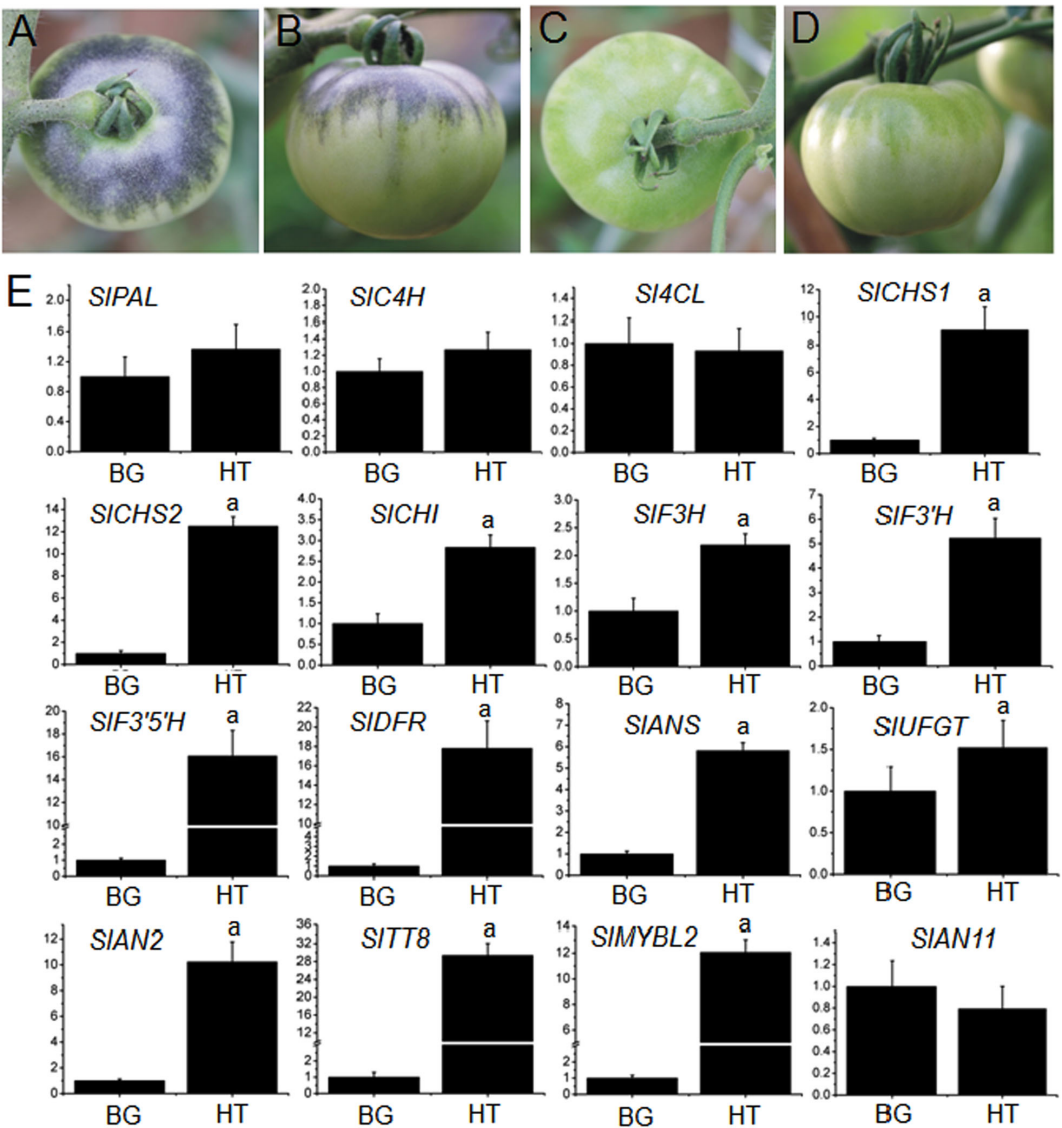
Anthocyanin accumulation and expression analysis of anthocyanin biosynthetic and regulatory genes in upper epicarps of *Pro35S:BrTT8* fruits under artificial high-light and low-light conditions. Anthocyanin accumulated specifically in epicarps near the stem end of transgenic fruits exposed to artificial high-light irradiance, **a** top view, **b** side view. Anthocyanin was not detected in epicarps near the stem end of transgenic fruits bagged with translucent plastic bags, **c** top view, **d** side view. **e** Expression profiles of anthocyanin biosynthetic and regulatory genes in upper epicarps of *Pro35S:BrTT8* fruits under artificial low-light and high-light conditions. The longitudinal axis indicates the expression levels of genes relative to *SlCAC*. BG fruits bagged with translucent plastic bags to simulate local low-light environments after anthesis, HT fruits exposed to high light. Biological replicates were performed in triplicate, and different lowercase letters indicate significance at *P* < 0.05

To further verify the hypothesis proposed above, *Pro35S:BrTT8* fruits were bagged with semitransparent bags immediately after pollination to simulate artificial low-light irradiance environments. Meanwhile, the whole plants were cultivated in a greenhouse under artificial high light. Compared with *Pro35S:BrTT8* fruits exposed to artificial high light, no anthocyanins were detected in the upper epicarps of the bagged fruits (Fig. 11 and Supplemental Figure S10). The fruits exposed to artificial high light showed intense purple/black pigmentation in the shoulder region, and the total anthocyanin content was approximately 0.36 mg g^−1^ FW in EMT, while the fruits covered with translucent plastic bags exhibited acyanic epicarps (Fig. 11 and Supplemental Figure S10). Transcripts of most anthocyanin structural genes, including *CHS1*, *CHS2*, *CHI*, *F3H*, *F3′H*, *F3′5′H*, *DFR* and *ANS*, of bagged EMTs were drastically less than those of EMTs exposed to high light (Fig. 11e). In addition, the low expression levels of anthocyanin activators, SlAN2 and SlTT8, further prove the inability of low-light irradiance to transcriptionally activate the expression of anthocyanin structural genes. Based on these results, we conclude that nonuniform anthocyanin pigmentation in *Pro35S:BrTT8* fruit epicarps is due to temporal and spatial expression of SlAN2 triggered by uneven high-light irradiance received by epidermal tissues. In addition, chlorophyll biosynthesis was clearly inhibited in fruits under artificial low-light conditions compared with fruits exposed to high light (Supplemental Figure S10).

## Discussion

In this study, *Pro35S:BrTT8* tomato plants were utilized to study light intensity effects on anthocyanin pigmentation, especially in reproductive tissues. High light could effectively trigger anthocyanin accumulation in both the leaves and fruits of *Pro35S:BrTT8* tomato plants. Moreover, anthocyanin pigmentation was uneven on the fruit surface, and the nonuniform distribution pattern was attributed to the direct sunlight flux received by epicarp tissue in the unit area of the fruit surface. Only in the epicarp cells that received a sufficient amount of light irradiance was the expression of the anthocyanin biosynthesis regulatory genes triggered, subsequently leading to enhanced expression of the anthocyanin biosynthetic genes and production of anthocyanins. Furthermore, it was demonstrated that anthocyanin pigmentation in both vegetative and reproductive tissues is mainly triggered by a heterogeneous MBW protein complex consisting of BrTT8, SlAN2 and SlAN11. Moreover, an R2R3-MYB repressor (SlMYBL2) can suppress the assemblage of the functional MBW complex by competitively binding with BrTT8, leading to the restricted production of anthocyanins. An excessive amount of anthocyanin production may serve as a signaling molecule to activate the expression of SlMYBL2, a competitive inhibitor of SlAN2, to maintain the balance between primary metabolism and secondary metabolism. To summarize all the findings in this study, a model that expands our understanding of the nonuniform anthocyanin pigmentation in tomato fruits under natural high-light conditions was proposed in Figs. 9 and 10.

### High-light-induced anthocyanin pigmentation in vegetative tissues of *Pro35S:BrTT8* plants was triggered by SlAN2

The light-induced expression of *BrTT8* was responsible for the intense purple pigments in bok choy^45^, and large amounts of anthocyanin pigmentation were only detected in *Pro35S:BrTT8* plants under high-light conditions. Meanwhile, anthocyanin pigmentation was observed mainly in leaf veins at a modest level in wild-type plants (Fig. 2e−i). In contrast, all tomato plants displayed an anthocyaninless phenotype under low-light conditions, regardless of their genetic background. Together, these results prove that light intensity has vital effects on anthocyanin accumulation in vegetative tissues of *Pro35S:BrTT8* tomato plants, according to the findings reported in transgenic petunia ectopically expressing Lc, a bHLH transcription factor that is homologous to BrTT8^35^. These results strongly suggest that the bHLH transgene alone is insufficient to induce anthocyanin biosynthesis effectively under low-light conditions. Therefore, it is reasonable to assume that BrTT8 may act with endogenous proteins induced by high light to initiate anthocyanin biosynthesis in *Pro35S:BrTT8* tomato plants. Therefore, both reported and putative direct regulators of anthocyanin biosynthesis were analyzed in the LC, HC, LT, and HT samples carefully. Most endogenous MYB regulators, including SlANT1, SlAN3 and SlAN4, SlAN11 (WD40 protein), SlGL3 (bHLH protein) and SlTRY (R3-MYB) were shown to be insensitive to changes in light intensity (Fig. 3b). The significant upregulation of SlAN2, SlTT8 and SlMYBL2 in both wild-type and *Pro35S:BrTT8* seedlings aroused our attention. In particular, the further enhanced expression of *SlTT8* in transgenic seedlings under high light indicates that it may be coordinately regulated by BrTT8 and other endogenous regulators (Fig. 3b), suggesting SlTT8 should not be a direct candidate for the regulator receiving transduction signals from high light.

Sequence analysis of MYB proteins suggests that SlAN2 may serve as a functional anthocyanin activator (Supplementary Figure S7 and S8). In addition, Y2H assays showed that BrTT8 could interact physically with endogenous regulators, including SlAN2, SlMYBL2 and SlAN11 (Fig. 7). Extensive studies have shown that anthocyanin biosynthetic genes are regulated directly by the MBW complex consisting of MYB, bHLH and WDR proteins^51,54–56^. Therefore, it was supposed that high-light-induced anthocyanin pigmentation in *Pro35S:BrTT8* seedlings was triggered by a heterogeneous MBW protein complex consisting of BrTT8, SlAN2, and SlAN11 (Fig. 9). As noteworthy production of anthocyanins also occurred in wild-type plants, an endogenous MBW protein complex consisting of endogenous transcription factors SlAN2, SlTT8, and SlAN11 may also contribute to the transcriptional activation of biosynthetic genes under high light. Moreover, the endogenous MBW protein complex might enhance anthocyanin pigmentation further in *Pro35S:BrTT8* tomato plants under high-light conditions. SlAN2 was demonstrated to have the ability to induce the biosynthesis of anthocyanins, phenolic acids and flavonols in infiltrated tobacco leaves, and BrTT8 could enhance the function of SlAN2. In contrast, SlMYBL2 significantly inhibited the accumulation of these secondary metabolites.

With the extensive evidence shown above, it was demonstrated that a heterogeneous MBW protein complex consisting of BrTT8, SlAN11 and SlAN2 triggered anthocyanin pigmentation in vegetative tissues of transgenic tomato plants under high light by transcriptional activation of structural genes (Fig. 9). Together with further enhanced expression of *SlTT8* in *Pro35S:BrTT8* plants exposed to high light, we may infer that *SlTT8* might be directly upregulated by the newly assembled heterogeneous MBW protein complex (Fig. 9). Moreover, the upregulated SlTT8 might interact physically with the endogenous regulators SlAN11 and SlAN2, resulting in the formation of an endogenous MBW complex (marked with a dashed cycle) and further enhanced anthocyanin production in *Pro35S:BrTT8* plants.

Sequence analysis showed that the [R/K]Px[P/A/R]xx[F/Y] motif conserved in MYB anthocyanin activators was absent from the C terminus of SlMYBL2^57^, in spite of an entire R2 domain at its N terminal region (Supplemental Figure S7). In addition, SlMYBL2 interacted with BrTT8 effectively in the Y2H assay. These results indicate that SlMYBL2 inhibits anthocyanin biosynthesis by competitively binding with the MYB interaction domain of BrTT8, preventing the formation of a functional MBW complex (Fig. 9). The dynamics of anthocyanin production and gene expression in *Pro35S:BrTT8* plants under high-light treatment prove that SlMYBL2 did not participate in the transcriptional regulation of anthocyanin structural genes in the early stages until the pigmentation intensity became high. Since too much anthocyanin accumulation can act as sunscreen to prevent light from being absorbed by chlorophyll pigments in leaves^35^, the induction of SlMYBL2 probably counterbalanced the high transcriptional activity of the MBW complex to avoid excessive anthocyanin production in a negative-feedback manner (Fig. 9). Since *PhMYB27*, an orthologous gene of *SlMYBL2*, was identified as a direct target of AN1 in petunia petals^26^, it can be speculated that SlMYBL2 might be transcription activated by the MBW complex itself under certain environments.

### Nonuniform anthocyanin pigmentation in fruits of *Pro35S:BrTT8* tomato plants triggered by region-specific expression of SlAN2 under high light

Delphinidin-based anthocyanins were identified as the main components in *Pro35S:BrTT8* tomato fruits (Table 1 and Supplemental Figure S1). In the flavonoid biosynthetic pathway, F3′5′H catalyzed the conversion of DHK to DHM by adding hydroxyl groups to the 3′ and 5′ positions on the B ring, leading to the synthesis of delphinidin-based anthocyanins (Fig. 1). Therefore, the drastic upregulation of SmF3′5′H was responsible for the abundant accumulation of delphinidin-3-rutinoside-5-glucoside in the epicarps of *Pro35S:BrTT8* plants. Furthermore, all anthocyanins identified in *Pro35S:BrTT8* fruits were glycosylated anthocyanidins that arise from glycosylation at the C5 and C3 positions of the core skeleton. From another perspective, these results prove that glycosylation was the main type of modification for anthocyanin precursors found in *Pro35S:BrTT8* tomato fruits.

Unlike vegetative tissues, anthocyanin pigmentation in *Pro35S:BrTT8* fruit exposed to natural high light was restricted in an approximately spherical area around the stem end. To further elucidate the unique pattern of anthocyanin accumulation, fruits were divided into four parts in wild-type and transgenic plants (Fig. 5b). The expression profiles of anthocyanin structural genes matched well with the pigment distribution patterns in fruits (Figs. 5c and 6). Apparently, the unique pattern of nonuniform anthocyanin pigmentation in fruits of *Pro35S:BrTT8* plants arises from region-specific expression of SlAN2 induced by natural high light. Similarly, the repressor SlMYBL2 was transcriptionally activated to prevent the excess production of anthocyanins. Herein, it seems that the molecular mechanisms underlying vegetative anthocyanin pigmentation are also suited for the coloration of *Pro35S:BrTT8* fruits under natural high light. However, it is not clear whether region-specific expression of SlAN2 arises from tissue specificity or high-light irradiance.

The results shown in Fig. 11 proved that intense purple pigmentation of the upper epicarps can also be induced efficiently by artificial high light. Moreover, transcripts of anthocyanin biosynthetic and regulatory genes in the upper epicarps of *Pro35S:BrTT8* fruits under both artificial low-light and high-light conditions were examined. It is possible that high light induced the high expression of SlAN2 in the upper epicarps, leading to the abundant assemblage of the MBW protein complex with BrTT8 and SlAN11 as cofactors. Ultimately, the region-specific enrichment of biosynthetic genes regulated by the MBW complex directly results in the unique distribution of anthocyanins in *Pro35S:BrTT8* fruits exposed to natural high light. However, even in the upper part of the epicarps, the pigment distribution was not uniform. To study this biological phenomenon thoroughly, a model describing the actual dose of light flux received by the fruit surface (epicarp tissue) under high light was proposed (Fig. 10). Generally, the equatorial lines of most fruits are parallel to the horizontal surface, and natural sunlight is approximately vertical to the surface. As the model shows, the actual value of luminous flux received in a unit area on epidermal tissue was only equal to the cosine *θ* proportion of direct sunlight passing through the same area on the horizontal surface. Therefore, the actual direct sunlight flux received in the unit area of epicarps decreased sharply with the enlargement of the angle *θ*. As a result, we speculate that light intensity below a certain value is insufficient to trigger visible anthocyanin production. As the unit area of the epicarps approaches the equatorial line, the actual dose of direct light received approaches zero, failing to induce visible anthocyanin accumulation. Logically, the pattern of direct sunlight intensity shed on the fruit surface coordinated well with the anthocyanin pigmentation strength. However, this model needs to be refined in future studies. Similarly, it was also reported that the green shoulder in AC results from a gradient pattern of expression of the plastid development regulator GLK2 throughout the fruit^53^. However, transcriptome profiling analysis revealed a broader gene expression gradient throughout the development of tomato fruits and rice leaves^53,58^. In terms of tissue patterning, several axes of developmental and morphological variation were proposed in fruit: apical-basal, medial-lateral, and abaxial-adaxial^59^. However, it is difficult to verify whether the gradient expression pattern of these genes arises from a gradient of tissue specificity from the stem end to the stylar end or from environmental factors. Altogether, the gradient of the sunlight flux received by epidermal cells at different latitudes on the fruit surface was responsible for the unique pattern of anthocyanin distribution in *Pro35S:BrTT8* epicarps exposed to natural high light.

Several positive regulators (SlAN2, SlANT1 and SlAN11) of anthocyanin accumulation in tomato plants have been characterized and investigated in previous studies^25,33,60,61^. However, the negative regulator ATV encodes an R3-MYB protein that can directly bind bHLH proteins and acts as a competitive inhibitor^33^. Recently, even the crosstalk between ethylene signal transduction and anthocyanin biosynthesis was studied^52^. However, nonuniform tomato fruit pigmentation under high light has not been studied. Remarkably, this article provides a good example of anthocyanin biosynthesis analysis in tomato fruits at metabolic and molecular levels under different light intensities. Furthermore, a model interpreting the nonuniform anthocyanin pigmentation of tomato fruits under natural high-light conditions was proposed. This article provides more insights into the biosynthesis and accumulation of nutritional substances in special environments and the genetic improvement of the market value of fruits and vegetables with future breeding.

## Supplementary information

Supplementary Figures 1–10

Supplementary Tables 1–4
