# Supplementary material for: Metabolic and molecular analysis of nonuniform anthocyanin pigmentation in tomato fruit under high light
Source: Hortic Res. 2019 May 1;6:56. doi: 10.1038/s41438-019-0138-2 (PMC6510810; doi:10.1038/s41438-019-0138-2)
Supplement: Supplementary file 1 — Supplementary Figures 1–10 [file 41438_2019_138_MOESM1_ESM.doc]

**
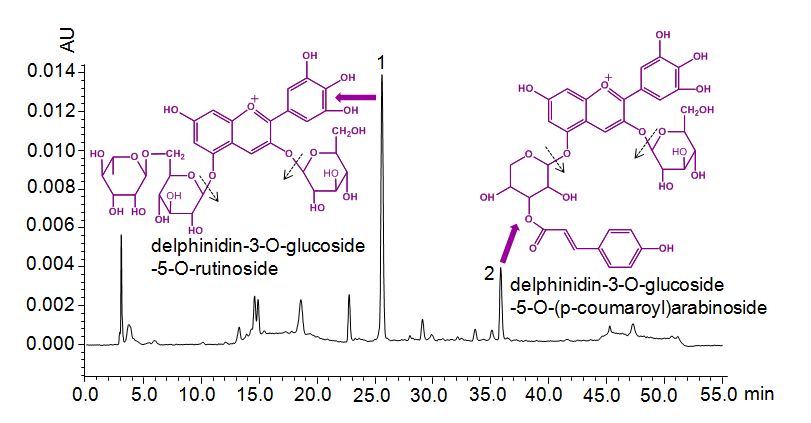
**

**Supplemental Figure** S1. HPLC profiles and structures of anthocyanins extracted from fruit epicarps of *Pro35S:BrTT8* plants grown under natural high-light conditions. Delphinidin-3-O-glucoside-5-O-rutinoside and delphinidin-3-O-glucoside-5-O-(p-coumaroyl)arabinoside referring to peak 1 and 2 respectively, are the main anthocyanins indentified by HPLC-ESI-MS/MS. The major cleavage sites confirmed by mass spectrometry were indicated with dashed arrows in the corresponding structures.

**
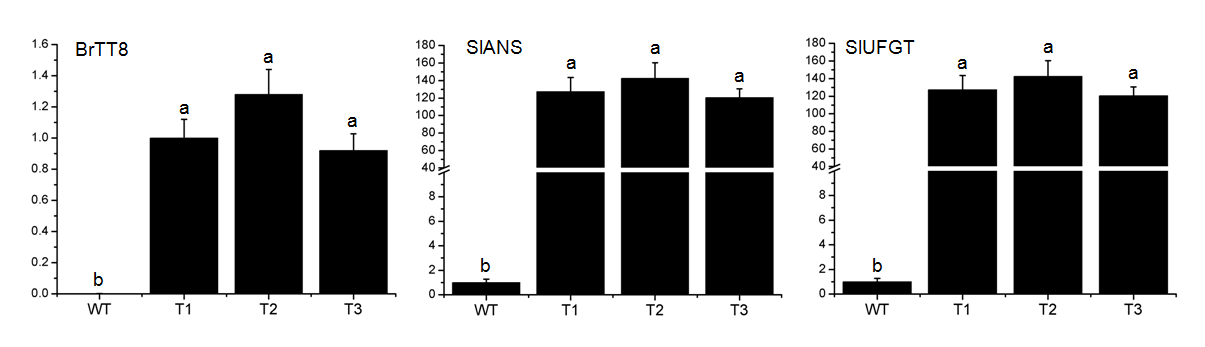
**

**Supplemental Figure** S2. Expression analysis of *BrTT8* and partial anthocyanin biosynthetic genes in leaves of wild-type and *Pro35S:BrTT8* plants grown under natural high-light conditions. T1, T2 and T3 indicate independent transgenic tomato lines. The longitudinal axis indicates the gene expression levels relative to *SlCAC*. Biological replicates were performed in triplicate and different letters indicate a significant difference at P ＜0.05.

**
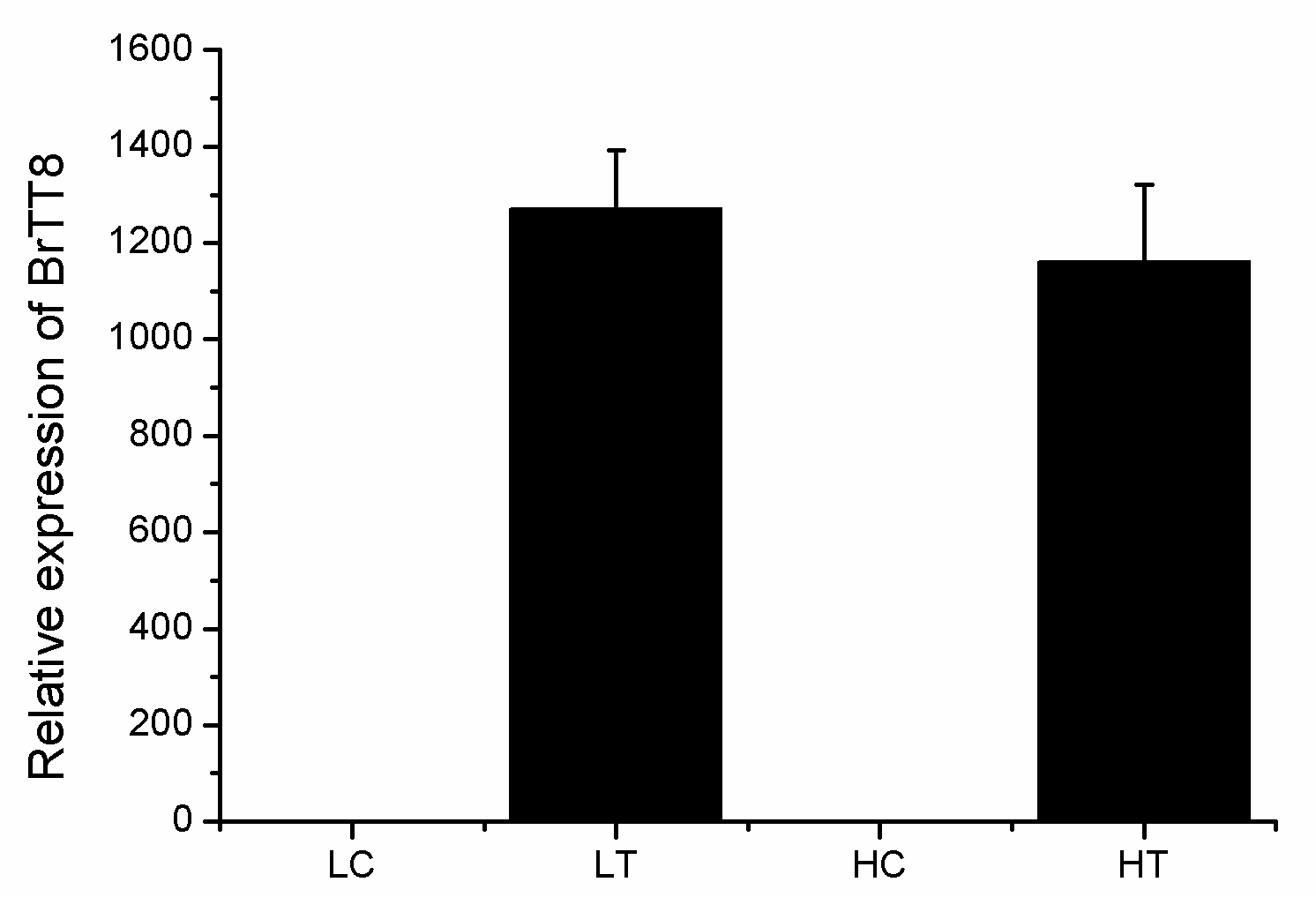
**

**Supplemental Figure** S3. Expression analysis of *BrTT8* in seedlings of wild-type and *Pro35S:BrTT8* plants grown under artificial high-light and low-light conditions. The longitudinal axis indicates the expression levels of *BrTT8* relative to *SlCAC*. LC: seedlings of control (wild type) under low-light conditions; HC: seedlings of control under high-light conditions; LT: seedlings of transgenic (*Pro35S:BrTT8*) plants under low-light conditions; HT: seedlings of transgenic plants under high-light conditions.

**
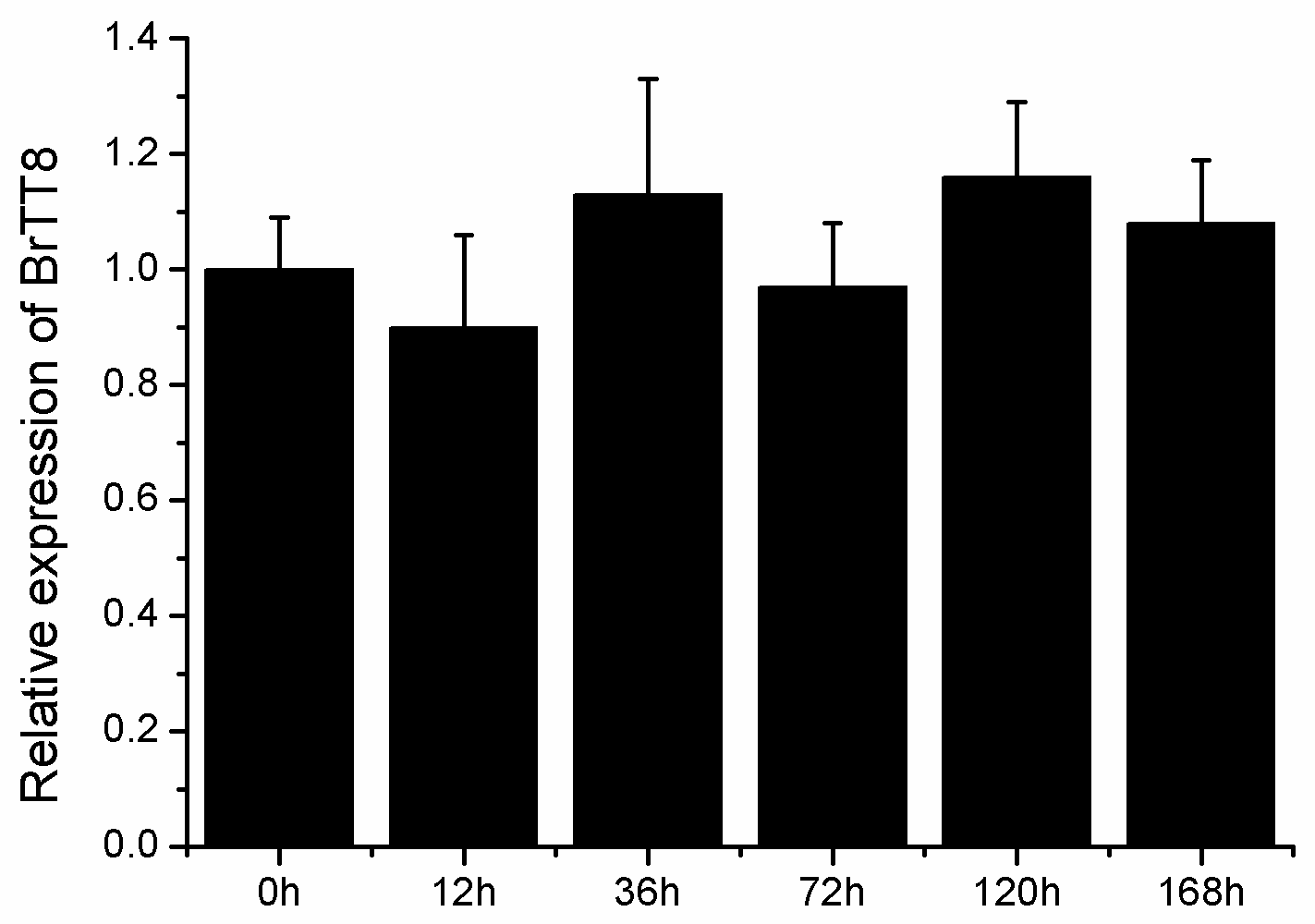
**

**Supplemental Figure S4.** Expression analysis of *BrTT8* in leaves of *Pro35S:BrTT8* tomato seedlings during artificial high-light treatment. The longitudinal axis indicates the expression levels of *BrTT8* relative to *SlCAC*.

**
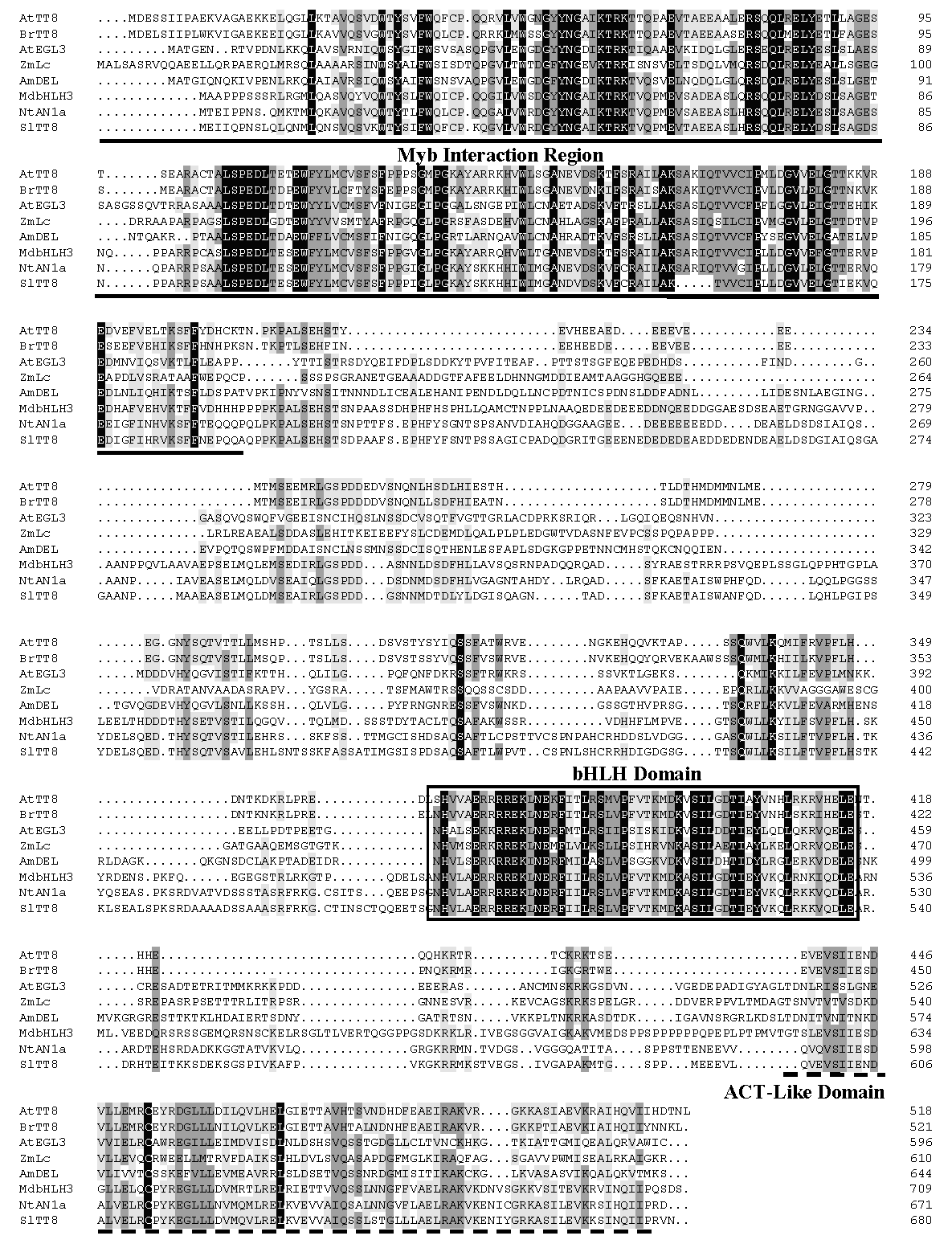
**

**Supplemental Figure** **S5**. A multiple sequence alignment of BrTT8 and SlTT8 with bHLH homologues from *Nicotiana tabacum* (NtAN1a), *Arabidopsis thaliana* (AtEGL3), *Zea mays* (ZmLc), *Antirrhinum majus* (AmDEL), *Malus domestica* (MdbHLH3) and *Arabidopsis thaliana* (AtTT8).

Clade I

Clade Ⅱ

**Supplemental Figure** S**6.** Phylogenetic reconstruction of BrTT8 and SlTT8 along with selected bHLHs using neighbor-joining method with MEGA 3.1 software. A minimum evolutionary phylogeny test and 1,000 bootstrap replicates were chosen for analysis. The scale bar represents 0.1 substitutions per site. Two clades are indicated by square brackets. The bHLH proteins, their respective plant species, and GenBank accession numbers are shown as follows: SlTT8, *Solanum lycopersicon* (Solyc09g065100.1.1); BrTT8, *Brassica rapa* (AIP94025); PhAN1, *Petunia x hybrid* (AAG25927); InbHLH1, *Ipomoea nil* (BAE94393); InbHLH2, *Ipomoea nil* (BAE94394); VvMYCA1, *Vitis vinifera* (ABM92332); VvMYC1, (ACC68685); AmDelila, *Antirrhinum majus* (AAA32663); PhJAF13 (AAC39455); AtGL3, *Arabidopsis thaliana* (NP_680372); AtEGL3 (NP_176552); AtTT8 (CAC14865); ZmIN1 (AAB03841); ZmLc (NP_001105339); OsRc, *Oryza sativa* (BAF42668); MdbHLH3 (ADL36597); NtAN1a (AEE99257); NtAN1b (AEE99258).


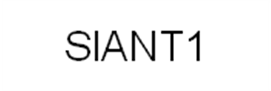

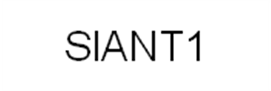

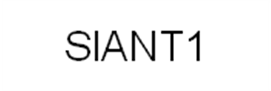


**R2 domain**

**R3 domain**

**Supplemental Figure S7.** An alignment of deduced amino acid sequences of MYB transcription factors from different species. The R2 and R3 domain are underlined with bold line and dashed line, respectively. The specific residues framed in red box represent the motif which interacts with bHLH co-factor in Arabidopsis. A C-terminal-conserved [R/K]Px[P/A/R]xx[F/Y] motif was framed in green box.


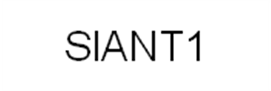


Clade I

Clade Ⅱ

Clade Ⅲ

**Supplemental Figure S8.** Phylogenetic analysis of deduced amino acid sequences of SlANT1、SlAN2、SlAN3、SlAN4 and SlMYBL2 along with selected MYB proteins. The phylogenetic tree was constructed using MEGA 3.1 with neighbor-joining method. Three clades are indicated by square brackets. A minimum evolutionary phylogeny test and 1,000 bootstrap replicates were chosen for analysis. The scale bar represents 0.3 substitutions per site. The MYB proteins and GenBank accession numbers are listed as follows: AtPAP1 (CAB09230); AtPAP2 (NP176813); AtMYB113 (NP176811); AtMYB114 (NP176812); SlANT1 (AAQ55181); PhAN2 (AAF66727); VvMYBA1, (AB242302); AmROSEA1, (ABB83826); MdMYB10 (ABB84753.1); BoMYB1 (ADP76649); BoMYB2 (ADP76650); SlANT1 (Solyc10g086260.1.1); SlAN2 (Solyc10g086250.1.1); SlAN3 (Solyc10g086270.1.1); SlAN4 (Solyc10g086290.1.1); SlTRY (Solyc01g095640.1.1) and SlMYBL2 (Solyc05g008250.1.1).


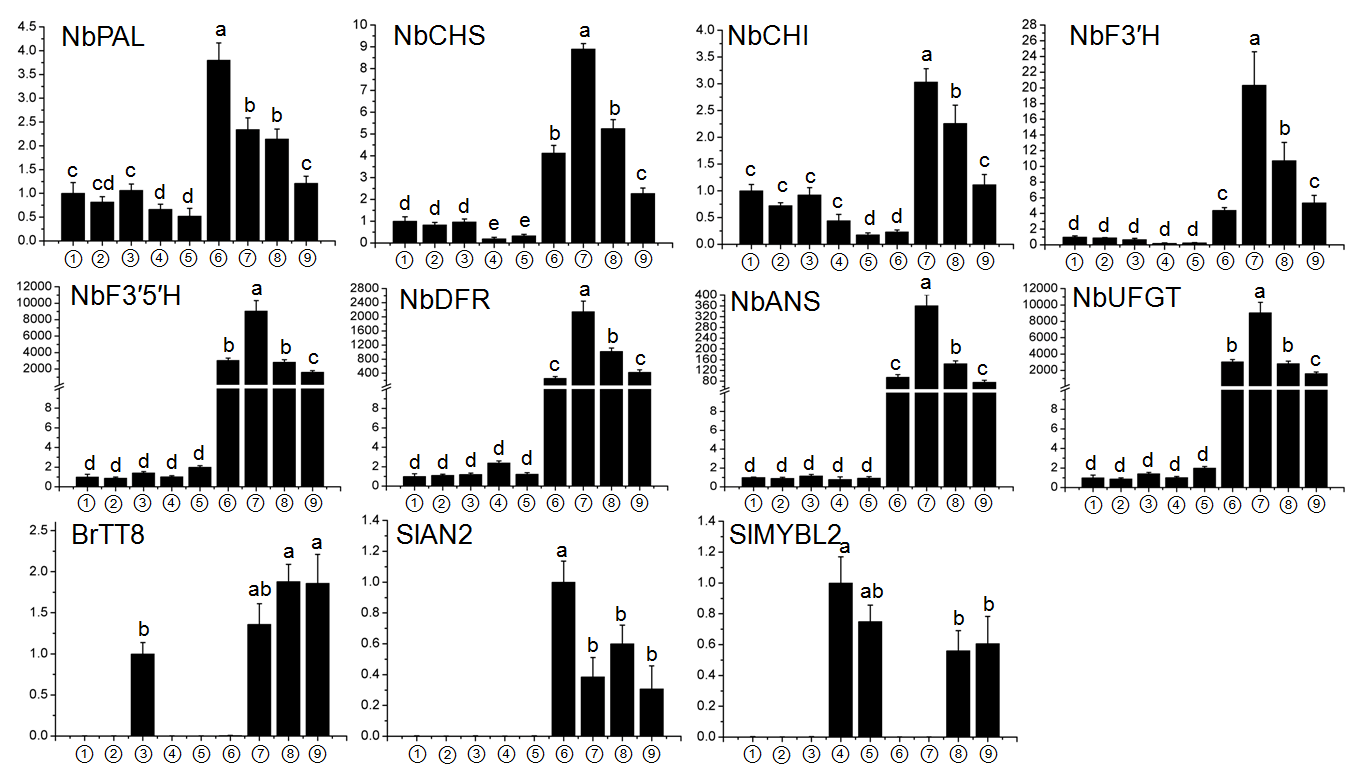


**Supplemental Figure S9.** Expression analysis of anthocyanin biosynthetic genes and exogenous genes in tobacco leaves infiltrated by *Agrobacterium tumefaciens* with different combinations of expression constructs. ①, ②, ③, ④, ⑤, ⑥, ⑦, ⑧ and ⑨ in reference to the samples analyzed in Figure 8 C. The longitudinal axis indicates the gene expression levels relative to *SlCAC*. Biological replicates were performed in triplicate and different lowercase letters indicate significance at P < 0.05.


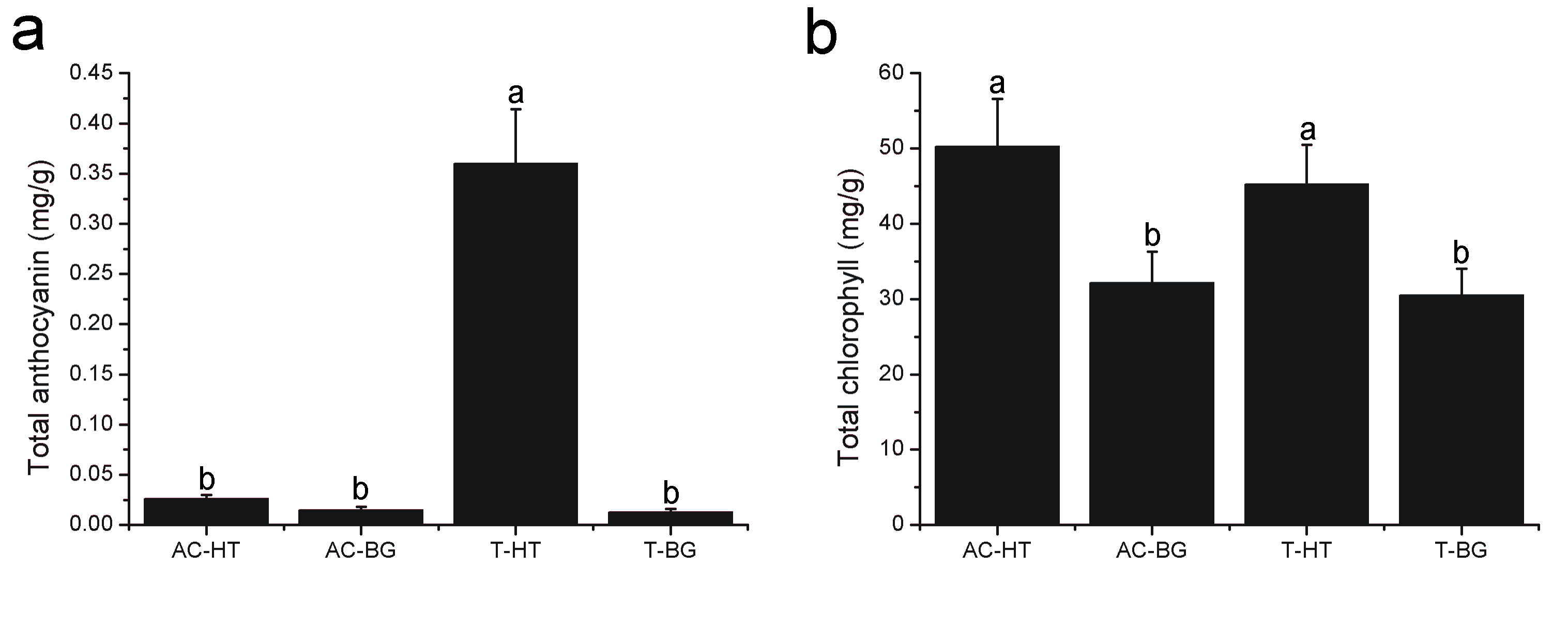


**Supplemental Figure S10.** Total contents of anthocyanins and chlorophyll pigments in epicarps of wild-type and *Pro35S:BrTT8* fruits under artificial high-light and low-light conditions. (a) Total anthocyanin contents in epicarps at stem end of fruits at mature green stage. (b) Chlorophyll contents in epicarps at stem end of fruits at mature green stage. Biological replicates were performed in triplicate and different letters indicate a significant difference at P ＜0.05. AC-HT: fruits of wild-type plants grown under artificial high-light conditions; AC-BG: bagged fruits of wild-type plants grown under artificial high-light conditions; T-HT: fruits of *Pro35S:BrTT8* plants grown under artificial high-light conditions; bagged fruits of *Pro35S:BrTT8* plants grown under artificial high-light conditions.
