# Supplementary material for: Metabolic and molecular analysis of nonuniform anthocyanin pigmentation in tomato fruit under high light
Source: Hortic Res. 2019 May 1;6:56. doi: 10.1038/s41438-019-0138-2 (PMC6510810; doi:10.1038/s41438-019-0138-2)
Supplement: Supplementary file 2 — Supplementary Tables 1–4 [file 41438_2019_138_MOESM2_ESM.doc]

**Supplemental Table S1.** Primers used for RT-qPCR analysis of both anthocyanin biosynthetic and regulatory genes in wild-type and transgenic tomato plants.

| Genes | Primer sets | Accession |
| --- | --- | --- |
| *SlPAL* | F: 5'-CGGTGAGGAGATTGATAAGG-3'  R: 5'-CATTTAGCAGATTGGAATAGGA-3' | TC233801 |
| *SlC4H* | F: 5'-CTAGCTAACAACCCCGCCCA-3'  R: 5'-AACTCCTCCTGCCAACACCG-3' | TC93956 |
| *Sl4CL* | F: 5'-GCTCTCTGCCCCGTAACCAA-3'  R: 5'-CCACGATGAAAAGCTCATCATCAT-3' | AK328438 |
| *SlCHS1* | F: 5'-CAAAAGAAGGCCTAGGAACTA-3'  R: 5'-CCCACTAAGCAGCAACACTG-3' | TC217706 |
| *SlCHS2* | F: 5'-GGGACCGTTATCCGACTGG-3'  R: 5'-GCTATCAGGGAGTAGAGTTTGG-3' | TC217840 |
| *SlCHI* | F: 5'-ACAGGGGCAGAACTAACTGAAAA-3'  R: 5'-TCAAAAACTTGTCAACTGGAGCA-3' | XM_004239923 |
| *SlF3H* | F: 5'-TGGCGATCACGGTCATTATTTG-3'  R: 5'-GCATCTGGTGCTGGATTCTGG-3' | HQ008775 |
| *SlF3’H* | F: 5'-CTGGTGGCAAGAAAGGTGG-3'  R: 5'-CGAATGGGGTATGAAAAGTAAGTC-3' | Solyc02g083860 |
| *SlF3’5’H* | F: 5'-ACTCCCGAGAGGTTCCTTAGC-3'  R: 5'-TCCACCACCACTATTCCCATC-3' | NM_001247911 |
| *SlDFR* | F: 5'-GCTGGAGCGATTTGGACTTC-3'  R: 5'-CAGCCTTCTCTGCCAGTATCTT-3' | Z18277 |
| *SlANS* | F: 5'-CTAAGCAACGGAAAGTACAAGAGC  R: 5'-CGGTGACAGTCTCAGGTAGGG | AJ785263 |
| *SlUFGT* | F: 5'-AGAGGCTGTGAAATTAGATAATGGG-3'  R: 5'-TACAACGGGAAGCTCGAGGG-3' | TC229189 |
| *SlANT1* | F: 5'-CAACGTAGATCCATGGTGGA-3'  R: 5'-GGGTGGTAAATTAAGAGAAAATTC-3' | Solyc10g086260 |
| *SlAN2* | F: 5'-TCAACCTCGGAACTTCTCAAA-3'  R: 5'-TTTCTCCTATTGGCTTCTCACA-3' | Solyc10g086250 |
| *SlAN3* | F: 5'-AAAACCTCAAGCTAGGAAGTTCA-3'  R: 5'-TGTTTTTCCATAATCAGTTACACCT-3' | Solyc10g086270 |
| *SlAN4* | F: 5'-GACCACGACCAAGACCAGG-3'  R: 5'-TTGTCCACCATTGAACTCCAT-3' | Solyc10g086290 |
| *SlMYBL2* | F: 5'-GATCCAAAAAATCATAGGCTATCT-3'  R: 5'-TGTCTAAATCAACCAGAGGACATA-3' | Solyc05g008250.1 |
| *SlTRY* | F: 5'-TCCCTCCTAATCAACAGCAAC-3'  R: 5'-TCTAATGAATTCCCACTCCATACT-3' | Solyc01g095640 |
| *SlTT8* | F: 5'-TTCCAAGTTTGCCTCTTCTGCTA-3'  R: 5'-CGACGACAGTGGGAGAGATTTG-3' | Solyc09g065100.1 |
| *SlGL3* | F: 5'-GATGGTGGTAGACGGTATTGGG-3'  R: 5'-CTCTTGACGGTCTAGTGGGCA-3' | AK320756 |
| *SlAN11* | F: 5-GTTGGCTTGGAACAAACAGG-3  R: 5-AGCAATAGCATTCACACTCGC-3 | Solyc03g097340.1 |
| *SlCAC* | F: 5'-TGGGTGTGCCTTTCTGAATG-3'  R: 5'-GCTAAGAACGATGGACCTAATG-3' | SGN-U314153 |
| *BrTT8* | F: 5'-CTACTCATCACGAGCCAAAC-3'  R: 5'-GCATCTCATCTCTAACAAAACAT-3' | KM403452 |

**Supplemental Table S2.** Primers used for constructing recombinant plasmids in yeast two-hybrid assay.

| Genes | Primer sets (5'to3') | note |
| --- | --- | --- |
| *BrTT8* | F: GCTCGAATTCATGGATGAATTAAGTATCAT  R: GATAGTCGACTAAGGTTAGAATCTCGGAA | pGBKT7-BrTT8 |
| *SlTT8* | F: GCTCGAATTCATGGAGATTATACAGCCTA  R: GATAGTCGACTTAATTAACTCTAGGGATTATC | pGBKT7-SlTT8 |
| *BrTT8* | F: GCTCGAATTCATGGATGAATTAAGTATCAT  R: GATACTCGAGTAAGGTTAGAATCTCGGAA | pGADT7-BrTT8 |
| *SlAN2* | F: GCTCGAATTCATGAATACTCCTATGTGTG  R: GATACTCGAGATTAAGTAGATTCCATAAGT | pGADT7-SlAN2 |
| *SlAN11* | F:GCTCGAATTCATGGAAAATTCAAGTCAAGAATC  R:GATACTCGAGCTCTAACAAAGAAAGACAGCCA | pGADT7-SlAN11 |
| SlMYBL2 | F: GCTAGGATCCATATGAGAAAGCCTTGTTGTG  R: GTAGCTCGAGATCGAGAATGTCTTCGATACT | pGADT7-SlMYBL2 |

**Supplemental Table S3.** Primers used for constructing recombinant plasmids in FRET assay.

| Names | Primer sequence (5' to 3') | note |
| --- | --- | --- |
| *EGFP-F1* | GCGTCTAGAATGGTGAGCAAGGGCGAG | EGFP |
| *EGFP-R1* | CCCGCGGTACCGTCGACGCTCCCTTGTACAGCTCGTCCAT | EGFP |
| *mCherry-F2* | CCCGGGATCCACCGGTCGCCACCATGGTGAGCAAGGGCGAG | mCherry |
| *mCherry-R2* | CCCGAGCTCCTACTTGTACAGCTCGTC | mCherry |
| *EGFP-R1-2* | GTGGATCCCGGGGGAGCGTCGACGGTACCGCGGG | EGFP-mCherry |
| *EGFP-R1-3* | CGGTGGATCCCGGGGGAGCGTCGACGGTACCGCGGGCT | EGFP-mCherry |
| *mCherry-F2-2* | CGTCGACGCTCCCCCGGG ATCCACCGGTCGCCACC | EGFP-mCherry |
| *mCherry-F2-3* | ACCGTCGACGCTCCCCCGGG ATCCACCGGTCGCCACCA | EGFP-mCherry |
| *SlAN2-mCherry-F1* | TATGGATCCATGAATACTCCTATGTGTGC | *SlAN2-mCherry* |
| *SlAN2-mCherry-R1* | CCCTTGCTCACCATAGTAGATTCCATAAGTCAATATCA | *SlAN2-mCherry* |
| *SlAN2-mCherry-F2* | TTATGGAATCTACTATGGTGAGCAAGGGCGAGGAGGAT | *SlAN2-mCherry* |
| *SlAN2-mCherry-R2* | ATTGAGCTCCTACTTGTACAGCTCGTC | *SlAN2-mCherry* |
| *BrTT8-EGFP-F1* | CTCTCTAGAATGGATGAATTAAGTATCATACCG | *BrTT8-EGFP* |
| *BrTT8-EGFP-R1* | CCCTTGCTCACCATGAGTTTATTATTATATATGATTTG | *BrTT8-EGFP* |
| *BrTT8-EGFP-F2* | ATAATAATAAACTCATGGTGAGCAAGGGCGAGGAGCTG | *BrTT8-EGFP* |
| *BrTT8-EGFP-R2* | ATAGAGCTC TTACTTGTACAGCTCGTCC | *BrTT8-EGFP* |

**Supplemental Table S4.** Primers used for RT-qPCR analysis of both anthocyanin biosynthetic and regulatory genes in infiltrated leaves of tobacco plants.

| Genes | Primer sets | Accession |
| --- | --- | --- |
| *NbPAL* | F: 5'-TGGGTGCCAATGGAGAACTT-3'  R: 5'-TGCAGGCGTCATCAGCGTAT-3' | Niben101Scf05442g03015 |
| *NbCHS* | F: 5'-CATTCCAACCATTAGGTCTTTCG-3'  R: 5'-TCACTCAACACTTGTCTTGTAGCC-3' | Niben101Scf02893g00001 |
| *NbCHI* | F: 5'-GGACAAACCTACCATTGCAGAA-3'  R: 5'-AAACAGAGCCTCCCACATTACA -3' | Niben101Scf05989g01008 |
| *NbF3H* | F: 5'-TCATCAAGCAGTGGTGAACTCG-3'  R: 5'-GGTCCTTACTCATTTTCCTCCTGT-3' | Niben101Scf09345g00004 |
| *NbF3’5’H* | F: 5'-CTTGATGTTGTTATGGCAAATAGGG-3'  R: 5'-GAAGAAGTGTCTGTACCAGCAGTG-3' | Niben101Scf14625g02006 |
| *NbDFR* | F: 5'- ACCGCCGCTGGTTGTTG-3'  R: 5'-GATGAATCTTCCCTCTGCCTTT-3' | Niben101Scf00305g05035 |
| *NbANS* | F: 5'- TGGGCGATTTTCTGTGAGC-3'  R: 5'-GCAAAGGTGCGAGGTGGG-3' | Niben101Scf00490g01007 |
| *NbUFGT* | F: 5'-GGCGTATCTTAGCTTTGGAACTG-3'  R: 5'-CCTTTAGGCAAAATCTTGACCC-3' | Niben101Scf05307g00001 |
| *NbACTIN* | F: 5-TCCGTTGCCCAGAAGTCC-3  R: 5-ACTGAGCACAATGTTACCGTAGAG-3 | Niben101Scf06087g02002 |
